# Extracellular vesicles as a promising biomarker resource in liquid biopsy for cancer

**DOI:** 10.20517/evcna.2021.06

**Published:** 2021-05-13

**Authors:** Takaaki Tamura, Yusuke Yoshioka, Shinichi Sakamoto, Tomohiko Ichikawa, Takahiro Ochiya

**Affiliations:** ^1^Department of Molecular and Cellular Medicine, Tokyo Medical University, Tokyo 160-0023, Japan.; ^2^Department of Urology, Graduate School of Medicine, Chiba University, Chiba 260-8670, Japan.

**Keywords:** Extracellular vesicles, liquid biopsy, cancer biomarker, microRNA

## Abstract

Liquid biopsy is a minimally invasive biopsy method that uses molecules in body fluids as biomarkers, and it has attracted attention as a new cancer therapy tool. Liquid biopsy has considerable clinical application potential, such as in early diagnosis, pathological condition monitoring, and tailored treatment development based on cancer biology and the predicted treatment response of individual patients. Extracellular vesicles (EVs) are lipid membranous vesicles released from almost all cell types, and they represent a novel liquid biopsy resource. EVs carry complex molecular cargoes, such as proteins, RNAs [e.g., mRNA and noncoding RNAs (microRNA, transfer RNA, circular RNA and long noncoding RNA)], and DNA fragments; these cargoes are delivered to recipient cells and serve as a cell-to-cell communication system. The molecular contents of EVs largely reflect the cell of origin and thus show cell-type specificity. In particular, cancer-derived EVs contain cancer-specific molecules expressed in parental cancer cells. Therefore, analysis of cancer-derived EVs might indicate the presence and nature of cancer. High-speed analytical technologies, such as mass spectrometry and high-throughput sequencing, have generated large data sets for EV cargoes that can be used to identify many candidate EV-associated biomarkers. Here, we will discuss the challenges and prospects of EV-based liquid biopsy compared to other biological resources (e.g., circulating tumor cells and cell-free DNA) and summarize the novel studies that have identified the remarkable potential of EVs as a cancer biomarker.

## INTRODUCTION

**Figure 1:**
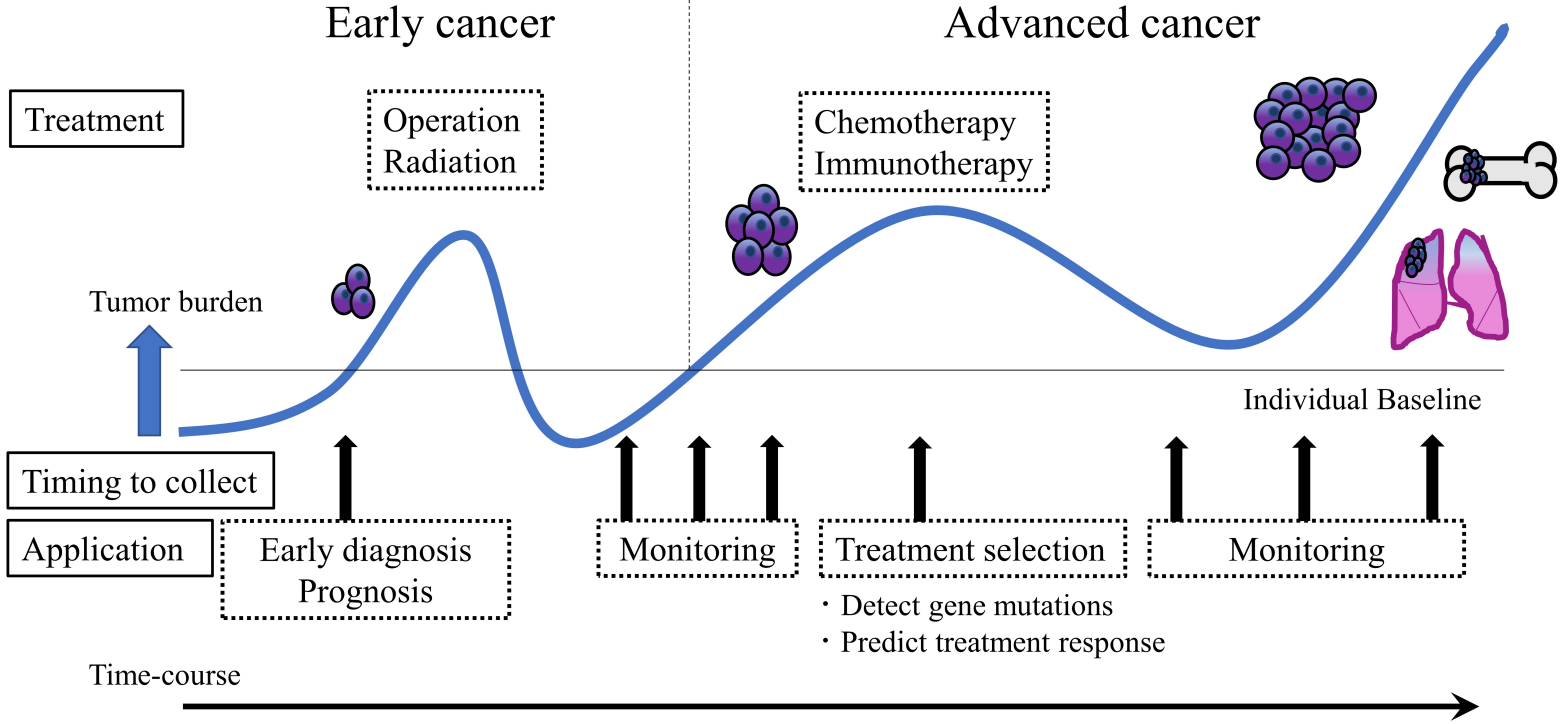
Clinical utility of liquid biopsy in cancer. Liquid biopsy presents a minimally invasive nature and thus has the potential to impact clinical practice at multiple stages of cancer management. This technique can contribute to early diagnosis, pathological condition monitoring, and tailored treatment development according to the cancer biology of individual patients. After cancer treatment, liquid biopsy can support follow-up care by providing early evidence of recurrence or therapy resistance.

Our body fluids, such as blood, urine, cerebrospinal fluid, saliva, pleural effusion, ascites fluid, breast milk and seminal plasma, contain various biological molecules^[1]^. Liquid biopsy is a minimally invasive method that uses these molecules as biomarkers, and it is emerging as a new tool in the strategy against cancer^[2]^. The terms "precision medicine"^[3]^ and "personalized medicine"^[4]^ have recently become popularized in the field of cancer research. Diagnosis and adequate therapy for individual cases of particular cancer types commonly rely on genetic mutation and gene expression analyses or pathological imaging observations of cancer lesions. However, such cancer genomic medicine approaches require collection of cancer tissue by biopsy, which imposes a heavy burden on the patient. In particular, it is difficult to obtain tumor tissue from organs located in the deeper parts of the body. Therefore, the development of minimally invasive methods, such as liquid biopsy, is desired. Liquid biopsy initially emerged only for the purpose of genetic diagnosis; however, it has considerable clinical application potential, such as in early diagnosis, monitoring of pathological conditions, and tailored treatment development according to cancer biology and the predicted treatment response^[5]^. Importantly, because of its minimally invasive nature, liquid biopsy can be scheduled more often to give more accurate snapshots of the disease at successive time points, which is useful for measuring temporal tumor burden levels and early evidence of recurrence or therapy resistance^[6] ^[Figure 1]. Furthermore, liquid biopsy may reflect the genetic profile of more cancer subclones in a patient than tissue biopsies, which are obtained from only one cancer region^[7]^.

Extracellular vesicles (EVs) have attracted increasing attention as a novel analyte in liquid biopsy^[8]^. EVs are lipid membranous vesicles that are released from almost all types of cells, including normal cells as well as abnormal cells, such as cancer cells. EVs are reported to be correlated with various biological phenomena and play important roles in cell-to-cell communication via horizontal transfer of cellular cargoes, such as proteins, RNAs [including mRNA and noncoding RNAs (e.g., microRNA; miRNA, transfer RNA; tRNA circular RNA; circRNA and long noncoding RNA; lncRNA)], DNA fragments, and lipids^[9,10]^. Importantly, the composition of EV cargoes secreted from individual cell types differs greatly depending on the cellular origin, and the characteristic features of EVs derived from various cancer cells have been revealed. Thus, cancer-derived EVs can be analyzed to determine the presence and nature of cancer. Furthermore, EVs have been reported to be found in almost all body fluids^[11]^. For these reasons, EVs are recognized as a promising liquid biopsy resource for cancer. In this review, we will discuss the feasibility and practicality of EV-based liquid biopsy in clinical settings. In the first half, we will argue the advantages and challenges of EV-based liquid biopsy for clinical application. In the second half, we will summarize recent notable studies investigating cancer-specific EV-related molecules as cancer biomarkers, with a focus on their biological or clinical significance.

## CANDIDATE ANALYTES IN LIQUID BIOPSY OTHER THAN EVS

**Figure 2:**
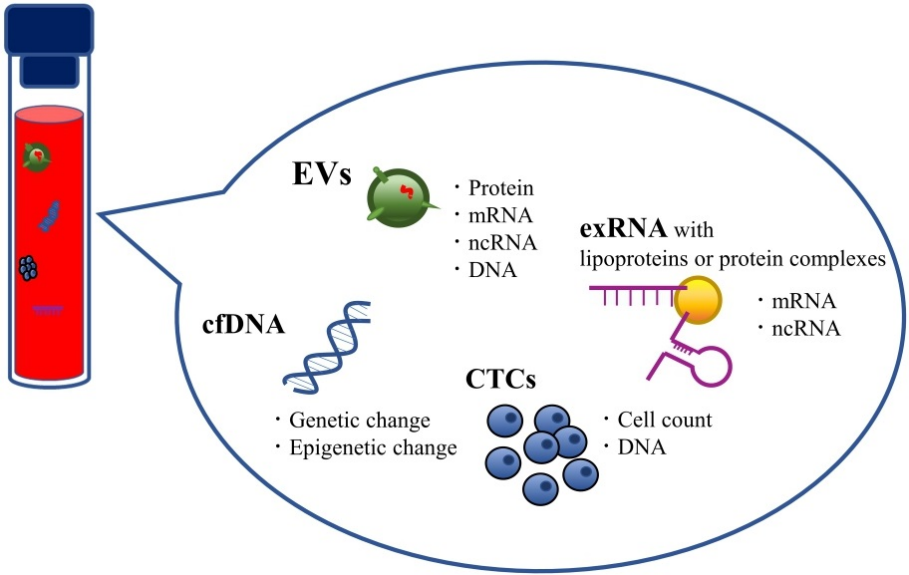
Candidate analytes of liquid biopsy. Body fluids contain several promising biomarkers for cancer. Each candidate analyte can provide considerable information about the cancer biology of individual patients.

Liquid biopsy can target various cancer-associated analytes in multiple body fluids, and several candidate analytes in liquid biopsy for cancer have been identified. These analytes include circulating tumor cells (CTCs) and circulating nucleic acids, such as cell-free DNA (cfDNA) and some extracellular RNA (exRNA) fragments [Figure 2]. Currently, CTCs and cfDNA are the most widely studied target materials for liquid biopsy^[12,13]^.

CTCs are cancer cells shed by primary or metastatic cancer lesions into the circulation and are considered a crucial determinant of hematogenous metastasis and recurrence. CTCs contain valuable information about the spreading tumor, and early detection of CTCs and treatment of metastatic spread can contribute to improving disease outcomes. Numerous studies in the past decade have shown that CTCs have potential as biomarkers to predict cancer metastasis progression and prognosis^[14,15]^. A high number of CTCs has been reported to be correlated with clinical outcome^[16-19]^. However, CTCs are estimated to account for at most one cell among a hundred million circulating cells; thus, there are generally only a small number of CTCs in a few milliliters of blood sample. Furthermore, CTCs must be analyzed as soon as possible after collection because the number of viable cells decreases rapidly. Therefore, analysis of CTCs requires relatively large volumes of fresh blood and advanced technology with extremely high analytical sensitivity and specificity^[20-22]^. To date, numerous technologies are available that are useful for enrichment and detection of CTCs^[23]^. Among them, the CellSearch^®^ system has received FDA approval for prognostic clinical evaluation of several cancer types^[24]^. However, due to the limited number of CTCs in the blood and the current level of detection technologies, CTCs have not yet been entirely accepted in the clinic. Research groups have recently moved towards analyzing CTC contents (e.g., miRNA^[25]^, mRNA^[26]^, and protein^[27,28]^) for detection of biomarkers.

cfDNA is a short fragment of nucleic acids found in body fluids, and a component of cfDNA derived from tumor cells is called circulating tumor DNA (ctDNA). The majority of ctDNA originates from apoptotic and necrotic tumor cells. Over the past decade, a large number of studies have reported that measuring ctDNA levels in cancer patients may help in cancer diagnosis and prognosis prediction^[29-31]^. Interestingly, ctDNA is reported to be horizontally transferred from cancer cells to normal cells via uptake of apoptotic bodies, which is one of the EV subclasses (described below), leading to cancer progression^[32,33]^. Furthermore, ctDNA is also reported to harbor genetic and epigenetic changes present in the original tumor, and analytical techniques to detect such changes have already been established. By examining these changes, researchers have shown that ctDNA also helps predict treatment response and recurrence^[34-38]^. The major challenge in ctDNA research is that tumor-specific mutations may only represent 0.01% of the total cfDNA^[39,40]^, thereby increasing the difficulty of detecting rare variants. Moreover, issues limiting cfDNA testing include its relatively short half-life^[41,42]^. Therefore, sample processing times are critical, and sample preservation requires special precautions, which are also significant barriers to practical use of cfDNA testing in the clinic.

CTC and ctDNA applications are confronted with several challenges, as described above. In short, the significantly low amounts and fragility of CTCs and ctDNA, which show remarkable variations in amount among individuals, increase the difficulty of detection. Moreover, CTC and ctDNA applications may be confined to evaluation of advanced cancer because CTCs are released from cancer tissue that has grown to the point that it causes metastases and ctDNA is primarily released from apoptotic or necrotic cancer tissue^[43]^. Thus, these entities may not be suitable for early diagnosis and may not reflect the current or near-future state of the disease.

exRNA refers to RNA that is present outside of cells within EVs or associated with platelets, lipoproteins and protein complexes. Similar to cfDNA, almost all non-EV-associated exRNAs are released into circulation by passive secretion. In addition to CTCs and cfDNA, non-EV-associated exRNA has also been well investigated as a candidate analyte in liquid biopsy for cancer. In particular, non-EV-associated miRNAs are recognized as a significant RNA subtype for biomarker discovery because miRNA profiles are associated with cancer-specific conditions. The critical issue is miRNA extraction from biofluid samples; the amounts of miRNA are highly variable among different experiments. Because of the small size of miRNAs and their attachment to other molecules, reproducible extraction remains an inherent issue^[44]^.

## WHAT ARE “EVS”?

We will now describe EVs, which are the main focus of this review. “EV” is a collective term that refers to all types of lipid membranous vesicles naturally released from all cell types. From the 1970s to the 1980s, membrane-enclosed vesicle structures were reported in various types of solid tissues, body fluids, and culture supernatants. Depending on their size and origin, these vesicles are called various names, such as prostasomes, exosomes, microvesicles, microparticles, shedding vesicles, ectosomes, apoptotic bodies and oncosomes. Each vesicle type contains slightly different molecular groups due to differences in vesicle biogenesis and secretion pathways. Nevertheless, their designations have been vaguely defined. Therefore, the International Society for Extracellular Vesicles (ISEV), founded in Sweden in 2012, recommends the use of “extracellular vesicle” as a generic term for these vesicles^[45,46]^. The nomenclature of these vesicles is detailed in the Minimal Information for Studies of Extracellular Vesicles (MISEV), which is the guideline advocated by the ISEV^[47,48]^.

EVs are classically classified into three main categories: exosomes (approximately 100 nm), microvesicles (approximately 1 µm), and apoptotic bodies (greater than 1 µm)^[49]^. These three classes of EVs differ in size as well as morphology, content, generation mode, and release mechanism. Exosomes are formed by the inward budding of early endosomes to form multivesicular bodies (MVBs). These MVBs fuse with the limiting plasma membrane to release exosomes into the extracellular space. Microvesicles originate by direct shedding or budding from the plasma membrane. Apoptotic bodies are released from cells undergoing programmed cell death^[50]^. Recently, Théry *et al*^[47,51]^. developed a more reasonable classification system for EVs and defined vesicles < 100 nm as small EVs, < 200 nm as medium EVs, and > 200 nm as large EVs. Moreover, they demonstrated that each EV subtype showed different characteristic protein components, suggesting that each EV category is generated and secreted through a specific molecular mechanism^[47,51]^.

## EVS AS AN ANALYTE IN LIQUID BIOPSY

In 1983, Pan and Johnstone^[52]^ discovered that cells secrete 100 nm-sized vesicles with a lipid membranous structure and that red blood cells release small vesicles loaded with the transferrin receptor, which is necessary to synthesize hemoglobin during maturation. They regarded EVs as “garbage bags” that are used to expel unused molecules from the cell and reported one of the remarkable EV functions; more importantly, this study demonstrated the presence of proteins in EVs^[52,53]^.

In the 1990s, by analyzing immune cells, Raposo *et al*.^[54]^ found that EVs affect recipient cells. EVs derived from B lymphocytes induced antigen-specific MHC class II-restricted T cell responses, indicating that EVs have functions related to intercellular communication^[54]^.

In 2007, Valadi *et al*.^[55]^ demonstrated that EVs from the human mast cell line HMC-1 and the mouse mast cell line MC/9 contain approximately 1300 mRNAs and 121 miRNAs and thus contribute to the exchange of genetic information between cells. Since that time, miRNAs have been the most widely studied class of short noncoding RNAs (ncRNAs) in EV cargoes^[55]^.

To date, EVs have been reported to contain various proteins, RNAs [including mRNAs and noncoding RNAs (miRNAs, tRNAs, circRNAs and lncRNAs)], and DNA fragments^[9]^. The past few years have seen extraordinary developments in areas such as mass spectrometry (MS), high-throughput sequencing (HTS) (e.g., DNA sequencing, chromatin immunoprecipitation sequencing, methylation sequencing, and RNA sequencing), and big-data analysis^[56,57]^. These advanced technologies allow us to select analytes contained in EVs and evaluate them deeply. Significantly, the composition of EV molecular contents reflects the intracellular status of their cellular origin. Moreover, these molecules are stably preserved due to their lipid-layered structure; hence, EVs from body fluids can be analyzed after being stored for a relatively long period of time^[58]^. A growing body of evidence has suggested that cancer-derived EV cargoes bare a strong resemblance to the intracellular status of their parental cells^[59]^. Analysis of EV cargoes can help reveal the existence, molecular profile and behavior of cancer. Thus, EV-based liquid biopsy contributes to early cancer diagnosis, monitoring of cancer pathological conditions, and treatment selection according to cancer biology and the predicted treatment response. In addition, EVs are easily accessible in various kinds of body fluids and appropriate for sequential collection. For these reasons, EVs have emerged as a promising biomarker resource and as another kind of liquid biopsy for cancer [Figure 2].

## CLASSICAL EV ISOLATION METHODS

Although EVs are a remarkable candidate analyte to detect cancer biomarkers, few EV-associated biomarkers have been implemented in clinical settings, which is partially due to the lack of adequate isolation methods^[60]^. In principle, EVs must be isolated from various biofluids for analysis. Indeed, in almost all cases, candidate EV-associated biomarkers were detected via isolation processes because target EV-associated molecules should be distinguished from EV-free molecules in the biofluid; however, a unified isolation method is not currently available, which is one of the biggest challenges in EV research.

The most popular technique for EV isolation is ultracentrifugation (UC), which separates EVs based on their size and buoyant density. The UC procedure does not require additional chemicals or pretreatment of the body fluid samples. Moreover, the combination of UC with other procedures, such as ultrafiltration, sucrose cushion, and density gradient centrifugation, can increase the purity of the EV fraction^[61-63]^. However, UC-based procedures generally require dedicated equipment and an extraordinary amount of time^[60]^. Moreover, repeated centrifugation can lead to reduced yield due to lost and aggregated EVs^[64]^. On the other hand, various polymer-based isolation kits, such as ExoQuick™, Total Exosome Isolation™ and miRCURY™, which are commercially available, can save time and labor costs, although issues with high contamination rates and high running costs have been reported^[63,65]^. An immunoaffinity capture approach using magnetic beads and monoclonal antibodies targeting EV surface antigens is also commonly used to isolate EVs^[66,67]^. Many reports have demonstrated that this approach achieves a selective and high-purity output; however, there are difficulties in terms of low capacity, low yield and high reagent cost^[60]^. Size-exclusion chromatography (SEC) is a technique for separating biological molecules based on molecular size in which target molecules are isolated by filtration through a resin-packed column. SEC has been reported to yield highly purified EVs and achieve excellent reproducibility in a relatively short time^[60,64]^; however, the existence of EVs in multiple fractions results in a low EV concentration in the obtained samples. Consequently, subsequent EV analysis often requires an additional concentration step^[68]^. Recently, many researchers have studied combined methods integrating multiple isolation techniques together or conducting them sequentially^[69]^. Among others, the combination of UC and SEC has demonstrated higher purity of EVs and better performance in subsequent analyses than the single-step methods^[70-72]^; however, in daily clinical settings, combined methods might be avoided due to several factors, such as time consumption, cost, repeatability, and ease of use. As high-quality EV samples are essential for subsequent analyses, there is an urgent need for a simple, cost-effective, and reliable technique for both basic research and clinical practice.

## EV-ASSOCIATED PROTEINS

### Common EV protein markers

EVs contain abundant proteins that reﬂect their origin and alterations of their parental cells. Based on the endosome-based biogenesis pathway, EV-speciﬁc protein markers include membrane trafficking-associated proteins (e.g., Rab family proteins, annexins), MVB-associated proteins (e.g., Alix, Tsg101 and ESCRT complex), tetraspanins (e.g., CD9, CD63 and CD81) and heat shock proteins (Hsp70 and Hsp90)^[73-75]^. As these proteins are common to EVs derived from almost all cell types, they can serve as common positive EV markers that can confirm the presence of EVs, and isolated EVs can be assessed via downstream proteomic analyses, such as western blotting (WB), enzyme-linked immunosorbent assay (ELISA), and flow cytometry (FCM), which use common EV proteins as hallmarks. With regard to checking the quality of isolated EV samples, MISEV recommends the use of apolipoproteins A1/2 and B and albumin as negative markers of blood-derived EVs because they are often co-isolated with these molecules^[47,76]^.

### EV protein-based platform for cancer diagnosis

Targeting membrane proteins on the surface of EVs is an effective strategy because target cancer-derived proteins can be directly detected without the use of a large sample volume or time-consuming isolation processes for EVs. Recently, great efforts have been devoted to establishing clinically useful detection platforms, including platforms for direct detection of cancer-specific EVs without any isolation or purification procedures. These platforms mainly consist of specific antibody-based technologies that detect EV surface proteins, such as ELISA.

Jørgensen *et al*.^[77]^ established the EV Array, which can detect EVs in unpurified materials in a high-throughput manner. The EV Array is composed of different capture antibodies located on a microarray slide, which capture EVs according to their surface proteins, and the target EVs are detected with a cocktail of biotinylated antibodies against the tetraspanins CD9, CD63, and CD81. The authors validated the performance of the EV Array by comparing plasma from nonsmall cell lung cancer (NSCLC) patients and normal healthy subjects^[78]^. Shao *et al*.^[79]^ reported that a microﬂuidic chip platform could distinguish patients with glioblastoma multiforme (GBM) from normal healthy subjects. This microﬂuidic chip labeled with magnetic nanosensors quantifies an EV-specific protein marker (CD63) and glioblastoma-speciﬁc proteins, such as epidermal growth factor receptor (EGFR) and EGFR variant III, on the surface of EVs via micronuclear magnetic resonance (μNMR). In this study, the authors also indicated that GBM EVs reﬂect gene ampliﬁcation or mutation and predict the therapy response. Surface plasmon resonance (SPR)-based nanosensors have recently attracted much attention due to their ability to detect a small number of molecules^[80]^. Im *et al*.^[81]^ developed an SPR-based exosome sensor called nanoplasmonic exosomes (nPLEXs). Each nanohole array of nPLEX is functionalized with antibodies that recognize EV surface proteins. nPLEX was able to differentiate ascites samples from ovarian cancer patients from healthy controls with an accuracy of 97% and identified ovarian cancer cell-derived EVs based on their expression of CD24 and EpCAM. Yoshioka *et al*.^[82]^ also established a highly rapid and sensitive analytical technique called “ExoScreen”. This assay consists of two kinds of antibodies against proteins on the surface of EVs that are detectable by photosensitizing beads. A very small sample volume (at least 5 μL) was required to detect EVs in serum from healthy controls without a complicated isolation process. Moreover, the assay could be completed within 2 h. In this study, they identified CD147 as a specific EV-surface protein derived from colorectal cancer cells and revealed that a larger number of CD9/CD147 double-positive EVs could be detected in serum from colorectal cancer patients than in serum from healthy control subjects using this assay. Furthermore, Zhao *et al*.^[83]^ developed a simple microﬂuidic platform named the “ExoSearch” chip that allows quantitative isolation of EVs using immunomagnetic beads. An “ExoSearch” chip could detect ovarian cancer by measuring three EV cancer protein markers, CA-125, EpCAM and CD24. The development of immune-capturing systems in microchips also provides highly sensitive and reliable detection of cancer markers without requiring a large sample volume or time-consuming EV isolation processes.

### Other EV protein biomarkers for cancer diagnosis

EV surface proteins and EV lumen proteins are regarded as candidate cancer biomarkers. Numerous studies have identified cancer-associated EV protein markers using isolation processes, followed by WB, ELISA, and FCM. Promising EV protein markers identified in clinical studies with patient body fluid samples are summarized in Table 1.

**Table 1:** A list of EV proteins as potential biomarkers for cancer

**Cancer types**	**Biological source**	**Isolation method**	**Detection method**	**Markers**	**Potential application**	**Ref.**
*Urinary cancer*						
Prostate cancer	Plasma	UC	ELISA	PSA	Diagnosis/Prognosis	[85]
	Urine	UC + SUC	ELISA/WB	PSA, PSMA	Diagnosis/Monitoring	[84]
	Plasma/Serum	UC/ExoQuick	ELISA/WB	Survivin	Diagnosis/Monitoring	[87]
	Urine	UC	IP/WB	δ-catenin	Diagnosis	[156]
	Serum	UC	WB	MDR-1/P-gp, MDR-3, PABP4	Predict chemoresistance (Docetaxel)	[95,157]
	Plasma	-	FCM	PSMA	Monitoring/Predict chemoresistance	[158]
Bladder cancer	Urine	UC	MS/WB	EH-domain-containing protein 4, EPS8L1, EPS8L2, GTPase NRas, Mucin 4, retinoic acid-induced protein3, resistin, alpha subunit of GsGTP binding protein	Diagnosis	[159]
	Urine	UC	ELISA	TACSTD2	Diagnosis	[160]
	Urine	UC	MS	α-1-anti-trypsin, H2B1K	Diagnosis	[161]
	Urine	UC	WB	HEXB, S100A4, SND1, TALDO1, and EHD4	Diagnosis	[162]
	Urine	UC	WB	Periostin	Diagnosis	[163]
	Urine	UC + SUC	WB	EDIL3	Diagnosis	[164]
	Urine	UC + SUC	FCM	CD36, CD44, 5T4, basigin, CD73, MUC1, α6-integrin	Diagnosis	[165]
Renal cancer	Urine	UC	MS/WB	MMP9, DKK4, EMMPRIN, CP, PODXL, CAIX, CD10, AQP1, dipeptidase 1, syntenin 1	Diagnosis	[166]
*Female cancer*						
Breast cancer	Serum	ExoQuick	ELISA	Survivin, Survivin2B	Diagnosis/Prognosis	[88]
	Serum/Plasma	ExoQuick	ELISA/WB	CD82	Diagnosis	[167]
	Ascites	UC + SUC	WB	CD24, EpCAM	Diagnosis	[168]
	Plasma	UC	FCM	TRPC5	Prognosis/Predict chemoresistance	[169]
	Serum	UC	FCM	UCH-L1	Predict chemoresistance (Anthracycline/taxan)	[92]
	Serum	UC	FCM/WB	HER2	Predict chemoresistance (Trastuzumab)	[96]
	Plasma	UC	ELISA/FCM	TGFβ1	Predict chemoresistance (Trastuzumab)	[97]
Ovarian cancer	Plasma	-	Exosearch chip	CD24, EpCAM, CA-125	Diagnosis	[83]
	Plasma	UC	WB	TGFβ1, MAGE3/6	Diagnosis	[170]
	Plasma	UC + SUC	WB	Claudin-4	Diagnosis	[171]
	Ascites	UC + SUC	WB	E-cadherin	Diagnosis/Prognosis	[172]
	Ascites	UC	WB	MMP2, MMP9, uPA	Diagnosis	[173]
	Ascites	UC + SUC	WB	CD24, L1CAM, ADAM10, EMMPRIN	Diagnosis/Prognosis	[174]
*Digestive cancer*						
Pancreatic cancer	Serum	UC	FCM	GPC1	Diagnosis/Prognosis	[89,175]
	Serum	UC + SUC	ELISA	CKAP4	Diagnosis	[176]
	Serum	UC	ELISA	MIF	Diagnosis/Prognosis	[177]
	Plasma	-	ELISA	EpCAM	Prognosis	[91]
Colorectal cancer	Serum	Exoquick	ELISA	CEA	Diagnosis	[178]
	Serum	-	ExoScreen	CD147, CD9	Diagnosis	[82]
	Ascites	UC	WB	claudin-3	Diagnosis	[179]
Gastric cancer	Serum	UC	FCM/WB	HER-2/neu, CCR6, EMMPRIN, MAGE-1, c-MET	Diagnosis	[180]
*Others*						
Lung cancer	Serum	UC	ELISA	EGFR	Diagnosis	[181]
(NSCLC)	Serum	UC	ELISA/WB	AHSG, ECM1 (with serum CEA)	Diagnosis	[182]
	Serum	-	EV array	30 Proteins	Diagnosis	[78]
	Plasma	-	EV array	CD171, NY-ESO-1, PLAP, Flotilin1	Diagnosis	[183]
	Urine	UC	WB	LRG1	Diagnosis	[184]
Melanoma	Plasma	UC	ELISA (Exo Test)/FCM/WB	Caveolin-1, CD63	Diagnosis/Prognosis	[185]
	Plasma	UC	WB	TYRP2, VLA-4, HSP70, HSP90	Prognosis	[186]
	Plasma	UC/Total Exosome isolation Kit	ELISA/FCM /WB	PDL-1	Predict immunotherapy resistance (Pembrolizumab)	[91]
Glioblastoma	Serum	-	µNMR system	EGFR, EGFRvⅢ, CD63	Prognosis	[79]

NSCLC: Non-small cell lung cancer; LSCC: laryngeal squamous cell carcinoma; UC: ultracentrifugation; SUC: sucrose cushion; ELISA: enzyme-linked immuno-sorbent assay; FCM: flow cytometry; WB: western blotting; IP: immunoprecipitation; MS: mass spectrometry; miRNA: microRNA; tRNA: transfer RNA; circRNA: circular RNA; lncRNA: long non-coding RNA.

Clinically validated traditional molecules have also been identified in EVs, such as prostate-specific antigen (PSA), and they represent novel diagnostic biomarkers. Mitchell *et al*.^[84]^ demonstrated that the expression of PSA and PSMA in urinary EVs can act as treatment response markers in prostate cancer. Additionally, Logozzi *et al*.^[85]^ showed increased PSA expression on EVs in vitro and in the plasma of prostate cancer patients. The authors emphasized the failure of current PSA testing in discriminating between benign prostatic hypertrophy and prostate cancer in terms of both overdiagnosis and overtreatment, which leads to patient suffering and public and private healthcare expenditures. Moreover, they claimed that EV PSA might resolve the problem associated with differences in PSA cutoff levels based on age, race and individual physiological condition. Similar to PSA, some traditional molecules in EVs have been discussed to have higher relevance to cancer than their total amount in body fluids, such as CEA for colon cancer^[86]^.

Additionally, some EV-associated markers have been reported as diagnostic markers for multiple cancer types. Khan *et al*.^[87]^ showed that Survivin, an inhibitor of apoptosis member, could be detected in plasma-derived EVs from both prostate cancer patients and healthy subjects; however, the relative amount of EV Survivin was remarkably higher in the plasma of prostate cancer patients. Their subsequent study showed that the EV Survivin and its alternative splice variants were also elevated in breast cancer patient plasma^[88]^, which suggests that EV Survivin might be an important diagnostic marker common to several cancer types.

### EV proteins can be used to monitor cancer progression and drug resistance

Biomarkers in EVs of several cancer types may be applied for cancer stratification because they change in response to anticancer therapy. Thus, EV proteins may be used as novel biomarkers to monitor cancer progression or to identify patients susceptible to anticancer drugs.

Melo *et al*.^[89]^ reported that glypican-1 (GPC1) could be a specific marker of cancer-derived EVs and that the existence of GPC1-positive EVs in serum could differentiate patients with pancreatic ductal adenocarcinoma (PDAC) from patients with benign pancreas disease or from healthy control subjects with 100% sensitivity and specificity. The levels of GPC1-positive EVs were significantly decreased after surgical resection; moreover, they were related to overall survival (OS) and significantly higher in patients with distant metastasis than in patients with lymph node metastasis only or no metastases^[89]^. Although this conclusion is controversial, a replication study was performed to validate this finding, and a subsequent discussion was held in ISEV2017^[90]^. Similarly, Giampieri *et al*.^[91]^ reported that a higher level of EpCAM-positive EVs before chemotherapy was correlated with shorter progression-free survival (PFS) and OS. In contrast, in this study, an increase in EpCAM-positive EV levels during treatment was correlated with better PFS in PDAC patients^[91]^. These studies demonstrated that EV-associated proteins could be biomarkers for monitoring tumor burden.

The development of chemoresistance is a persistent problem during cancer treatment. Various studies have reported cell-to-cell transfer of multidrug resistance (MDR) efflux pumps as EV cargoes from chemotherapy-resistant cells to chemotherapy-sensitive cancer cells. EVs from doxorubicin-resistant^[92]^ or docetaxel-resistant^[93]^ breast cancer cell lines transferred chemoresistance to recipient cancer cells through P-glycoprotein (P-gp) loaded onto EVs. Moreover, the same phenomenon in paclitaxel-resistant ovarian cancer cells was also caused by the transfer of functional P-gp mediated by EVs^[94]^. Docetaxel-resistant prostate cancer cells were also reported to proliferate through cell-to-cell transfer of EV P-gp. In this study, serum EVs from prostate cancer patients who were nonresponders to docetaxel therapy protected prostate cancer cells from the cytotoxicity of docetaxel^[95]^. These studies indicate that EV-P-gp might be a promising marker to predict chemotherapy resistance in several cancer types. Ubiquitin C-terminal hydrolase L1 (UCH-L1) has also been reported as an EV-based predictive biomarker of chemoresistance in breast cancer. UCH-L1 overexpression has been reported to induce upregulation of P-gp levels through the MAPK/ERK signaling pathway, thereby enhancing an MDR phenotype in breast cancer. Ning *et al*.^[92]^ showed that higher UCH-L1 levels in circulating EVs are correlated with poorer response to adjuvant anthracycline/taxane-based chemotherapy. In addition, they demonstrated that UCH-L1-positive EVs derived from breast cancer cell lines could transfer chemoresistance to recipient cells in vitro, indicating that EVs might be a predictive biomarker of chemoresistance in breast cancer patients.

Interestingly, EV-mediated drug resistance has been reported to be relevant for molecular target drugs as well as chemotherapy drugs. Ciravolo *et al*.^[96]^ reported that 73% of advanced-stage breast cancer patients had HER2-positive EVs in circulation, which hampered the corresponding therapeutic efficacy of trastuzumab monoclonal antibody. Importantly, this study demonstrated that the presence of HER2-positive EVs in the serum of breast cancer patients could be an indicator to predict a patient’s response to trastuzumab therapeutic regimens^[96]^. Martinez *et al*.^[97]^ reported that EVs released from HER2 drug-resistant cells contain larger amounts of the immunosuppressive cytokine TGF-β1. Importantly, a recent neoadjuvant clinical trial by the same group that included trastuzumab and lapatinib further demonstrated that HER2-overexpressing breast cancer patients who were nonresponders to HER2 drug therapy had significantly higher amounts of TGF-β1 in circulating EVs than patients who did not respond to HER2 drug therapy. These results suggest that EV-TGF-β1 can be a biomarker for predictive response to this therapeutic regimen against breast cancer^[97]^.

Recently, with the successful development of immune checkpoint inhibitors (ICIs), cancer immunotherapy has attracted worldwide attention as a new cancer treatment. EV proteins may contribute to identifying patients susceptible to ICIs. Tumor cells avoid immune recognition by upregulating the surface expression of programmed death-ligand 1 (PD-L1), which interacts with the programmed death-1 (PD-1) receptor on T cells to elicit the immune checkpoint response. Indeed, immunotherapy with anti-PD-1 antibodies has shown remarkable therapeutic effects against different tumor types^[98]^. However, for some patients, the therapeutic response has been reported to be rather poor^[99,100]^. To address this problem, Chen *et al*.^[101]^ showed that specific EVs reduce the effectiveness of immunotherapy approaches in certain patients with melanoma. Remarkably, using human melanoma xenografts in nude mice, they showed that metastatic melanoma cell lines release EVs loaded with PD-L1 on their surface and that interferon-γ increases the expression of PD-L1 on these vesicles, which suppresses the function of CD8+ T cells, facilitating tumor growth. Importantly, these authors showed that the level of PD-L1-positive EVs differentiates responders from non-responders to anti-PD-1 therapy. More importantly, this study provides evidence supporting the application of PD-L1-positive EVs as a predictive biomarker for anti-PD-1 therapy in melanoma patients^[101]^.

## EV-ASSOCIATED NUCLEIC ACIDS

As described above, high-speed analytical technologies, such as HTS, are powerful tools for identifying candidate nucleic acid biomarkers. For detection and validation of candidate EV nucleic acid biomarkers, reverse transcription-quantitative polymerase chain reaction (RT-qPCR) after an isolation process is the most common detection method. Recently, digital polymerase chain reaction (dPCR) has emerged as a novel detection method for EV nucleic acids. dPCR is a qPCR technique that provides a sensitive and reproducible method of measuring the amount of DNA or RNA present in a sample^[102]^. The tremendous progress in these technologies during the last few decades has allowed us to analyze EV-associated nucleic acids derived from diverse body fluids.

### EV RNA biomarkers

Pioneering studies of nucleic acids from isolated EVs have identiﬁed various miRNAs and mRNAs as the major components of EVs^[55,103]^. Subsequently, several important papers reporting on the function of EV-miRNAs were published and showed that the transferred EV-miRNAs can be active in recipient cells and modify the cellular phenotype^[104-106]^. Over the last decade, studies have revealed that EVs contain other noncoding RNAs, such as tRNAs^[107]^, circRNAs^[108]^ and lncRNAs^[109,110]^. To date, EV RNAs (as EV cargo) have received the most attention in terms of cancer diagnosis and prognosis because they are easy to quantify using conventional methods, such as qPCR, and stable against RNase-dependent degradation in the circulatory system^[111]^. Table 2 summarizes the candidate EV RNA markers reported as diagnostic and prognostic tools for cancer.

**Table 2:** A list of EV RNAs as potential biomarkers for cancer

**Cancer types**	**Biological source**	**Isolation methods**	**Detection methods**	**RNA types**	**Markers**	**Potential application**	**Ref.**
*Uninary cancer*							
Prostate cancer	Urine	UC + SUC	nestedPCR	mRNA	PSA, PCA-3, TMPRSS:ERG	Diagnosis/Monitoring	[115]
	Urine	-	ExoDx^®^ Prostate (IntelliScore)	mRNA/lncRNA	PCA3, ERG, SPDEF	Diagnosis	[118]
	Urine	UF/UC	RT-qPCR	miRNA	let-7c, miR-21, miR-107, miR-145, miR-196a-5p, miR-204, miR-375, miR-501-3p, miR-574-3p, miR-2909	Diagnosis	[116,187-191]
	Serum/Plasma	UF/SEC/UC	RT-qPCR	miRNA	let-7i, miR-16, miR-21-5p, miR-24, miR-26a, miR-26b, miR-30c-5p, miR-34b, miR-92b, miR-93, miR-103, miR-106a, miR-107, miR-130b, miR-141, miR-181a-2, miR-195, miR-197, miR-200c-3p, miR-210-3p, miR-223, miR-298, miR-301a, miR-326, miR-328, miR-331-3p, miR-346, miR-375, miR-432, miR-574-3p, miR-625, miR-1290, miR-2110	Diagnosis	[187,192-196]
	Plasma	UF/SEC/UC	RT-qPCR	miRNA	miR-17, miR-20a, miR-23a, miR-130b, miR-198, miR-200b, miR-375, miR-379, miR-513a-5p, miR-572, miR-577, miR-582-3p, miR-609, miR-619, miR-624, miR-1236, miR-1290	Prognosis	[119,187]
	Serum	UC	RT-qPCR	miRNA	miR-1246	Prognosis	[197]
	Urine	-	Urine Exosome RNA Isolation Kit / RT-qPCR	lncRNA	p21	Diagnosis	[122]
	Plasma	Total Exosome Isolation Kit	RT-qPCR	lncRNA	SAP30L- AS1, SChLAP1	Diagnosis	[198]
	Plasma / Urine	-	exoRNeasy kit/ddPCR	mRNA	AR-V7	Predict hormone therapy resistance (Abiraterone and/or Enzalutamide)	[132,133]
Bladdar cancer	Urine	UC	ddPCR	miRNA	miR-21, miR-93, miR-200c, miR-940	Diagnosis	[199]
	Urine	-	ddPCR	miRNA	miR-21-5p, miR-4454, miR-720, miR-200c-3p, miR-29b-3p, miR-200b-3p	Diagnosis	[200]
	Urine	UC	RT-qPCR	miRNA	miR-375, miR-146a	Diagnosis	[201]
	Urine	unknown	RT-qPCR	miRNA	miR-146b-5p, miR-155-5p	Diagnosis	[202]
	Urine	UC	RT-qPCR	lncRNA	HOTAIR, HOX- AS-2, MALAT1, SOX2, OCT4	Diagnosis/Prognosis	[203]
	Serum	ExoQuick	RT-qPCR	lncRNA	PCAT1, UBC1, SNHG16	Diagnosis/Prognosis	[204]
	Serum	UC	RT-qPCR	lncRNA	UCA1	Diagnosis	[205]
	Urine / Serum	UC	RT-qPCR	circRNA	PRMT5	Diagnosis/Prognosis	[126]
Renal cancer	Serum	UC + IP	RT-qPCR	miRNA	miR-210, miR-1233	Diagnosis	[206,207]
	Urine	UC	RT-qPCR	miRNA	miR-150-5p, miR-126-3p in combination with miR-34b-5p, miR-449a and miR-486-5p	Diagnosis	[208]
	Serum	Total Exosome Isolation Kit	RT-qPCR	miRNA	miR-224	Prognosis	[209]
	Plasma	ExoQuick	RT-qPCR	miRNA	miR-26a-1-3p, miR- let-7-I, miRN-615-3p	Prognosis	[210]
	Urine	UC	RT-qPCR	lncRNA	GSTA1, CEBPA, PCBD1	Diagnosis	[211]
*Female cancer*							
Breast cancer	Plasma	FC/UC/ExoQuick	RT-qPCR	miRNA	miR-21, miR-1246	Diagnosis	[212]
	Plasma	UC	RT-qPCR	miRNA	miR-105	Diagnosis	[213]
	Serum	unknown/Total Exosome Isolation Kit	RT-qPCR	miRNA	miRNA-21, miRNA-222, miRNA-155	Prognosis/Predict chemoresistance (miR-21, miR-155: Doxorubicin, Paclitaxel)	[214,215]
	Serum	UC + IP	RT-qPCR	miRNA	miR-200a, miR-200c, miR-205	Diagnosis	[216]
	Bood/Milk/Ductal fluids	UC	RT-qPCR	miRNA	miR-16, miR-1246, miR-451, miR-205	Diagnosis	[217]
	Serum	ExoQuick	RT-qPCR	lncRNA	HOTAIR	Prognossis/Monitoring	[129]
	Serum	ExoQuick	RT-qPCR	lncRNA	SNHG14	Predict chemoresistance (Trastuzumab)	[130]
	Plasma	UC	RT-qPCR	mRNA	TrpC5, mdr1, MUC1 and flotillin2	Predict chemoresistance (Anthracycline/taxan)	[135]
	Serum	Total Exosome Isolation Kit	RT-qPCR	mRNA	GSTP1	Predict chemoresistance (Anthracycline/taxan)	[136]
Ovarian cancer	Serum	UC + IP	RT-qPCR	miRNA	miR-21, miR-141, miR-200a, miR-200c, miR-200b, miR-203, miR-205, miR-214	Diagnosis	[112]
	Serum	Total Exosome Isolation Kit	RT-qPCR	miRNA	miR-1246	Predict chemoresistance (Paclitaxel)	[218]
*Digestive cancer*							
Pancreatic cancer	Serum/Urine	UC	RT-qPCR	miRNA	miR-17-5p, miR-21	Diagnosis	[219]
	Plasma	ExoQuick	RT-qPCR	miRNA	miR-155	Predict chemoresistance (Gemcitabine)	[131]
	Saliva	UC	RT-qPCR	mRNA	Apbb1ip, ASPN, Daf2, FoxP1, Bco31781, Gng2	Diagnosis	[220]
Liver cancer (HCC)	Plasma	Total Exosome Isolation Kit	RT-qPCR	tRNA	ValTAC-3, GlyTCC-5, ValAAC-5, GluCTC-5	Diagnosis	[107]
	Serum	Exoquick	RT-qPCR	circRNA	circUHRF1	Diagnosis/immunotherapy resistance (anti-PD-1)	[108]
	Serum	Exoquick	RT-qPCR	miRNA	miR-21, miR-10b	Prognosis	[221]
Gastoric cancer	Serum	unknown	RT-qPCR	miRNA	HOTTIP	Diagnosis/Prognosis	[124]
	Serum	UC	RT-qPCR	lncRNA	lncUEGC	Diagnosis	[123]
	Serum	Exoquick	RT-qPCR	circRNA	circSHKBP1	Diagnosis	[222]
Esophageal cancer (ESCC)	Serum	Exoquick	RT-qPCR	miRNA	miR-21	Diagnosis	[120]
Colorectal cancer	Serum	UC	RT-qPCR	miRNA	let-7a, miR-1229, miR-1246, miR-150, miR-21, miR-223, miR-23a	Diagnosis	[127]
	Serum	ExoQuick	RT-qPCR	miRNA	miR-4772-3p	Prognosis	[223]
Rectal cancer	Plasma	miRCURY Exosome Isolation Kit	RT-qPCR	miRNA	miR-30d-5p, miR-181a-5p and miR-486-5p	Diagnosis	[224]
*Others*							
Lung cancer	Plasma	ExoQuick	RT-qPCR	miRNA	miR-151a-5p, miR-30a-3p, miR-200b-5p, miR-629, miR-100, and miR-154-3p	Diagnosis	[225]
Lung cancer (NSCLC)	Plasma	IP	RT-qPCR	miRNA	let-7f, miR-20b, miR-30e-3p, miR-223, miR-301	Diagnosis	[226]
Lung cancer (LSCC)	Plasma	ExoQuick	RT-qPCR	miRNA	miR-205, miR-19a, miR-19b, miR-30b, miR-20a	Monitoring	[227]
Lung cancer (NSCLC)	Serum	UC	RT-qPCR	miRNA	miRNA-222-3p	Prognosis/Predict chemoresistance (Gemcitabine)	[228]
Lung cancer (NSCLC)	Serum	ExoQuick	RT-qPCR	miRNA	miRNA-146a-5p	Predict chemoresistance (Cisplatin)	[229]
Lung cancer (NSCLC)	Plasma	-	exoRNeasy kit/ddPCR	mRNA	PD-L1	Predict immunotherapy resistance (anti-PD-1)	[134]
Melanoma	Plasma	-	exoRNeasy kit/ddPCR	mRNA	PD-L1	Predict immunotherapy resistance (anti-PD-1)	[134]
Glioblastoma	Serum/Cereblospinal fluid	UC	RT-qPCR	miRNA	miR-21	Diagnosis/Prognosis	[230]
	Cereblospinal fluid	UC	RT-qPCR	miRNA	miR-21	Diagnosis	[231]
	Serum	ExoQuick	RT-qPCR	miRNA	RNU6-1, miR-320, miR-574-3p	Diagnosis	[232]
	Serum	-	iMER	mRNA	MGMT, APNG	Predict chimoresistance (Temozolomide)	[137]

HCC: Hepatocellular carcinoma; ESCC: esophageal squamous cell cancer; NSCLC: non-small cell lung cancer; LSCC: laryngeal squamous cell carcinoma; UC: ultracentrifugation; SUC: sucrose cushion; UF: ultrafiltration; IP: immunoprecipitation; RT-qPCR: reverse transcription-quantitative PCR; ddPCR: droplet digital PCR; miRNA: microRNA; tRNA: transfer RNA; circRNA: circular RNA; lncRNA: long non-coding RNA.

### EV RNAs for cancer diagnosis

Since the discovery of EV-miRNAs by Valadi *et al*.^[55]^ in 2007, numerous studies have been performed to identify diagnostic EV miRNAs for cancer. Taylor *et al*.^[112]^ showed that eight miRNAs (miR-21, miR-141, miR-200a, miR-200c, miR-200b, miR-203, miR-205 and miR-214) that were previously characterized as diagnostic markers for ovarian cancer were also upregulated in circulating EVs derived from ovarian cancer patients. Rabinowits *et al*.^[113]^ conducted miRNA-proﬁling analyses of EV-based liquid biopsy samples and tumor biopsy samples from lung cancer patients and healthy controls. Their study showed a similarity in miRNA patterns between EV-based biopsy samples and tumor biopsy samples from lung cancer patients and demonstrated significant differences between these miRNA patterns and those in EVs from the healthy control group. This result indicates the potential of EV miRNAs as a liquid biopsy source for lung cancer. On the other hand, EV miR-1246 was signiﬁcantly increased in ESCC patient serum samples but was not elevated in tumor biopsy samples, indicating that the level of EV miRNAs may not necessarily reﬂect the amounts in parental cells^[114]^.

Regarding EVs in other body ﬂuids, Nilsson *et al*.^[115]^ reported that PCA3 and TMPRSS2:ERG mRNA, which are previously established prostate cancer biomarkers, were also present in urinary EVs from prostate cancer patients. Additionally, Foj *et al*.^[116]^ demonstrated that the levels of miR-21-5p, miR-375, and let-7c-5p were remarkably increased in urinary EVs derived from prostate cancer patients compared with those from healthy subjects. Analyses of urinary EVs and associated RNAs are difficult to perform because of the low abundance of these molecules and the fact that the urine volume itself varies greatly over time. However, recently, a new technology has been reported in which zinc oxide nanowires are used to catch urinary EVs to increase the yield^[117]^. Moreover, ExoDx^®^ Prostate (IntelliScore) is a simple, non-DRE, urine-based test for prostate cancer that is commercially available in the United States. This test has been clinically validated for risk stratification of clinically significant prostate cancer (Gleason score ≥ 7) from low-grade prostate cancer (Gleason score 6) and benign prostate disease, thus avoiding unnecessary prostate biopsy. A patient-specific individual risk score is evaluated based on an original algorithm that combines the expression level of three RNAs (PCA3 noncoding RNA, ERG mRNA, and SPDEF mRNA), which are correlated with clinically significant prostate cancer, detected directly in urinary EV RNAs^[118]^.

To date, the potential of many miRNAs as cancer prognostic markers has been reported. Huang *et al*.^[119]^ showed that increased levels of serum miR-1290 and miR-375 in EVs were correlated with decreased OS in patients with advanced-stage prostate cancer. Tanaka *et al*.^[120]^ revealed that high levels of circulating EV miR-21 could distinguish esophageal squamous cell cancer (ESCC) patients from patients with benign diseases. The levels of EV miR-21 were also correlated with cancer progression and aggressiveness, indicating that EV miR-21 can serve as a diagnostic biomarker for cancer as well as a therapeutic target. In addition to EV miR-21, EV miR-1246 was also identiﬁed as a diagnostic and prognostic marker for ESCC^[114]^. Regarding prognostic markers for digestive cancers, Matsumura *et al.*^[121]^. reported that serum EV miR-19a-3p might be a prognostic biomarker to predict the recurrence of colorectal cancer. Other noncoding RNAs, such as lncRNAs and the less common family of circRNAs in EVs from cancer cells, have also attracted increasing attention. Urinary EV lncRNA p21 was reported to be elevated in prostate cancer patients and able to distinguish prostate cancer patients from those with benign disease^[122]^. Lin *et al*.^[123]^ reported that lncRNA upregulated in plasma exosomes from gastric cancer (lncUEGC1) patients could be used to detect early-stage gastric cancer. Serum EV-associated lncRNA HOTTIP was also reported as a diagnostic and prognostic indicator of gastric cancer^[124]^. Furthermore, Lee *et al*.^[125]^ demonstrated the prognostic significance of circulating EV ncRNAs [miRNA-21 and lncRNA activated by transforming growth factor beta (lncRNA-ATB)] for human hepatocellular carcinoma. In this study, the OS and PFS rates were signiﬁcantly lower in patients with higher levels of miR-21 and lncRNA-ATB in EVs^[125]^. Concerning circRNA, Chen *et al*.^[126]^ showed that circRNA PRMT5 was highly enriched in both serum and urinary EVs collected from patients with bladder cancer compared with normal individual cohorts. In this study, they also revealed that EV circPRMT5 levels were significantly correlated with cancer metastasis^[126]^.

### EV RNAs to monitor cancer progression and drug resistance

Similar to EV proteins, EV-RNAs have been reported to have potential as biomarkers for monitoring therapeutic effects or resistance to anticancer therapy.

Ogata-Kawata *et al*.^[127]^ reported that the levels of seven miRNAs (let-7a, miR-1229, miR-1246, miR-150, miR-21, miR-223, miR-23a) in serum EVs were signiﬁcantly increased in colorectal cancer patients. The serum levels of these miRNAs were signiﬁcantly decreased after tumor resection, indicating their potential for monitoring tumor burden^[127]^. Svedman *et al*.^[128]^ investigated EV-associated miRNA levels in patients with metastatic melanoma before, during, and after MAPK inhibitor therapy. They showed that increased levels of let-7g-5p were correlated with lower tumor burden and could clearly differentiate responders from nonresponders; moreover, an increase in the miR-497-5p level during the treatment course was significantly associated with better PFS rates^[128]^. Several researchers have investigated EV-associated lncRNAs in patients during chemotherapy. HOTAIR is one of the major lncRNAs that is overexpressed in a variety of cancers and promotes cancer cell proliferation, invasion and migration. Tang *et al*.^[129]^ reported that EV HOTAIR was more enriched in serum samples from breast cancer patients than in healthy controls. In addition, they showed that a high pretreatment level of EV HOTAIR was correlated with a poor response to neoadjuvant chemotherapy and tamoxifen hormone therapy. In this study, EV HOTAIR was significantly decreased in all patients after surgery compared with before surgery, indicating that serum EV HOTAIR originates from the tumor tissue, and its level is associated with tumor burden and cancer aggressiveness^[129]^.

Regarding EV lncRNAs, Dong *et al*.^[130]^ have previously shown that isolated EVs from HER2-positive advanced breast cancer patients who were nonresponders to trastuzumab therapy contained more lncRNA SNHG14 than EVs from responders. In this study, the authors showed that EV lncRNA-SNHG14 promoted trastuzumab resistance by activating Bcl-2/apoptosis regulator BAX (Bax) signaling. Similar to this study, other types of EV RNAs have also been reported to be involved in resistance to anticancer therapy. Mikamori *et al*.^[131]^ reported that higher miR-155 expression levels in resected tumor tissue samples from pancreatic ductal adenocarcinoma patients treated with gemcitabine (GEM) were correlated with a poorer prognosis. This study demonstrated that the levels of miR-155 in plasma-derived EVs were consistent with those in pancreatic tissue^[131]^. The results suggest that circulating EV biomarkers can reflect, to some extent, the tumor burden and possess potential as real-time monitoring biomarkers for anticancer drug resistance. Interestingly, they further demonstrated that high expression of miR-155 in pancreatic cancer cell lines promoted antiapoptotic signaling and EV secretion in vitro. Moreover, they showed that the EVs released by miR-155-overexpressing PDAC cell lines could transfer chemoresistance-associated molecules (including miR-155) to other cancer cells and that the recipient cells subsequently acquired chemoresistance to GEM in vitro. Del Re *et al*.^[132]^ succeeded in detection of androgen receptor splice variant 7 (AR-V7) in EV RNAs from castration-resistant prostate cancer (CRPC) patients. The authors showed that EV AR-V7 mRNA levels were associated with hormonal therapy resistance. OS was significantly shorter among patients with EVs containing AR-V7 mRNA than among those without AR-V7 mRNA present (3 months *vs*. 20 months)^[132]^. Woo *et al*.^[133] ^reported a practical method for analysis of AR-V7 mRNA in urinary EVs. In this study, the authors adopted a lab-on-a-disc integrated with six independent nanofiltration units, which enabled simultaneous processing of six individual samples. EV mRNA was extracted from each 4 mL urine sample, and AR-V7 and androgen receptor full-length (AR-FL) mRNA levels were quantified by dPCR. The results showed that higher AR-V7 and lower AR-FL expression was detected in urinary EVs derived from patients with CRPC than in those from patients with hormone-sensitive prostate cancer. In addition, they described that AR-V7 transcript levels and the AR-V7/AR-FL ratio in urinary EVs were higher in patients with advanced prostate cancer^[133]^. These studies indicate that EV AR-V7 mRNA could be a predictive biomarker for hormonal therapy resistance in prostate cancer. Some studies have investigated the relevance between EV RNAs and the response to ICIs. For example, Del Re *et al*^[134]^. demonstrated that EV-associated PD-L1 mRNA levels could be useful for real-time monitoring of the response to anti-PD-1 antibody therapy. They evaluated PD-L1 mRNA expression levels in plasma EVs from melanoma and NSCLC patients during nivolumab and pembrolizumab therapy. After 2 months of treatment, the EV PD-L1 mRNA copy numbers were significantly decreased in responders but remained unchanged in nonresponders^[134]^. Regarding the EV-based biomarkers associated with breast cancer chemoresistance, Ma *et al*.^[135]^ performed profiling analyses of mRNA in circulating EVs from breast cancer patients treated with chemotherapy or not. The authors demonstrated that four mRNAs (TrpC5, MDR1, MUC1, and flotillin2) were amplified in EVs from patients with chemotherapy but were not amplified in patients without chemotherapy^[135]^. Interestingly, considering that TrpC5 was previously shown to regulate P-gp expression in recipient cells, this study suggested that TrpC5-enriched circulating EVs could promote cancer MDR development in these patients. More recently, Yang *et al*.^[136]^ analyzed the levels of glutathione S-transferase P (GSTP1) mRNA in serum EVs from breast cancer patients treated with anthracycline/taxane-based neoadjuvant chemotherapy. GSTP1 is an enzyme that has a critical role in cell detoxification. Importantly, they observed that patients with EVs highly enriched in GSTP1 mRNA showed an inadequate response to chemotherapy^[136]^.

Shao *et al*.^[137]^ established a microfluidic platform termed immunomagnetic exosome RNA (iMER) analysis, which integrates immunomagnetic selection targeting EV-surface proteins and real-time qPCR for collecting EV-associated RNAs into a single microfluidic chip form. Serial measurements of the mRNA levels of MGMT and APNG, two important enzymes involved in repairing DNA damage induced by temozolomide for glioblastoma, demonstrated the feasibility of drug resistance monitoring during treatment using this integrated platform^[137]^. Although this kind of novel technology for capturing and analyzing EVs requires further development and validation in a clinical setting, it has the potential to reach the next level of EV utilization to detect cancer biomarkers.

### EV DNA biomarkers

In addition to RNA species, DNA fragments have also been identiﬁed in EVs. Balaj *et al*.^[138]^ were among the first to show that EVs contain single-stranded DNA. Yokoi *et al*.^[139]^ confirmed the presence of double-stranded DNA in EVs using imaging flow cytometry and described how nuclear content was loaded into EVs. EV DNA is also a promising diagnostic tool due to its ability to carry information regarding cancer-speciﬁc mutations^[140]^. Here, we aim to summarize the growing evidence for EV DNA as a diagnostic marker and to consider its diagnostic advantages compared to cfDNA.

Kahlert *et al*.^[141]^ identiﬁed the genomic DNA fragments in EVs from pancreatic cancer cell lines and pancreatic cancer patients. All genomic sequencing revealed mutations in KRAS and p53 in the genomic DNA of EVs derived from pancreatic cancer, suggesting that EV DNA sequencing can be used to determine treatment plans and predict therapy resistance^[141]^. However, in a subsequent paper, the first author called into question the superiority of EV DNA profiling to cfDNA profiling as an analyte for liquid biopsy^[142]^. Another remarkable study showed the detection rate of KRAS mutations in circulating EVs in PDAC patients and healthy controls. Mutations were detected in 7.4%, 66.7%, 80%, and 85% of age-matched controls and localized, locally advanced, and metastatic PDAC patients, respectively. In this study, the mutant KRAS detection rate in patients with localized PDAC after surgical resection dropped from 66.7% before surgery to 5% after surgery, indicating that EV-associated KRAS mutations could serve as biomarkers for real-time monitoring of therapy response and tumor burden^[143]^. A subsequent study demonstrated that the kinetics of EV-associated KRAS mutant allele frequency (MAF) were deeply correlated with neoadjuvant chemotherapy response; 71% of patients with a lack of cancer progression showed decreased KRAS MAF, while 94% of patients with cancer progression showed no decrease in KRAS MAF^[144]^. Interestingly, whereas the KRAS mutation detection rate in localized and metastatic pancreatic cancer was nearly equivalent for the cfDNA and EV DNA analyses, surgically resected primary tissue samples showed 95.5% concordance with the EV DNA-based assessment and only 68.2% concordance with the cfDNA analysis results. Similarly, agreement between the results for pancreatic patient tissue and liquid biopsy analyses was reported to be 83.3% for EV DNA-based analysis and only 66.8% for cfDNA-based analysis.

Similar to KRAS mutations, several analyses of circulating EVs from NSCLC patients showed the presence of clinically relevant epidermal growth factor receptor (EGFR)-specific mutations^[145,146]^. Remarkably, these studies highlight the expanded performance associated with combining analysis of EV-associated nucleic acids together with cfDNA *vs*. cfDNA analysis alone. Castellanos-Rizaldos *et al*.^[145]^ reported that the combined use of EV nucleic acids together with cfDNA overcame the limited abundance of the EGFR T790M mutation and other EGFR mutations and contributed to improved sensitivity and specificity compared to cfDNA alone. Krug *et al*.^[146]^ showed that the combined use of EV RNA and cfDNA sequencing improved the detection of EGFR mutations up to 98% *vs*. 84% for cfDNA alone. EV-based liquid biopsy has potential for multiplexing DNA analyses with analyses of other EV cargoes, such as miRNA, lncRNA, and proteins; thus, it can provide highly accurate information about cancer biology.

Other body ﬂuids, such as urine, may also be valuable sources for EV DNA-based liquid biopsies. Lee *et al*.^[147]^ investigated whether genetic alterations in urothelial bladder cancer were reﬂected in urinary cfDNA or EV DNA and demonstrated concordance between the copy number proﬁles of tumor tissue and urinary DNA (cfDNA and EV DNA), with allelic frequencies of 56.2% and 65.6%, respectively. Ampliﬁcation of MDM2, ERBB2, CCND1, and CCNE1 and deletion of CDKN2A, PTEN, and RB1, whose alterations are all frequently found in bladder cancer, were also identiﬁed^[147]^.

In addition to these studies, many studies have indicated that EV DNA may have a significant impact on the origin cells and recipient cells by playing a role in the maintenance of cellular homeostasis^[148]^ and acting as an intercellular messenger^[149]^. Furthermore, similar to EV RNA, the packaging of DNA into membrane-enclosed vesicles contributes to enhanced stability by protecting it from the external environment and avoiding recognition by the immune system^[150]^. These findings may demonstrate that EV DNA is superior to cfDNA as a biomaterial in liquid biopsy for cancer; however, current protocols for definitively detecting cancer-derived EV DNA in clinical samples are hampered by high labor costs, high financial costs, and low accuracy. The ctDNA isolation and detection method has already been established and might be used in routine clinical situations in the near future^[142]^. Although cfDNA was first reported in the 1940s^[151]^, the presence of EV DNA has long been doubted and was only demonstrated in the 2010s^[138,140]^. The potential of EV DNA as an analyte for liquid biopsies has not been thoroughly investigated but is a promising research area. We must improve strategies to utilize EV DNA and establish more useful methods of applying this molecule for liquid biopsy.

## CHALLENGES AND FUTURE PERSPECTIVE

Most potential biomarkers are based on small-scale studies and require longitudinal validation in larger samples. In addition, the consistency and variability in data collected using different technologies are significant problems. The large amounts of data from recently developed detection technologies provide an opportunity to identify key molecules but also represent a challenge to differentiating valuable markers among numerous candidate molecules, and the associated processes need further investigation. Deeper and more rigorous studies are required to accurately correlate these potential markers with clinical practice. Moreover, the basic knowledge regarding the biological characteristics of EVs is still insufficient. The mechanisms that regulate the heterogeneity of cancer EVs have not been fully elucidated, and the factors that affect EV synthesis, secretion and transfer remain poorly understood; overcoming these issues is crucial to improving the accuracy of EV diagnostic outcomes.

The future of EV-based liquid biopsy depends on meeting certain technological issues. Isolation and detection of EVs are undoubtedly the biggest problems mentioned above; however, other technological problems have been observed. For example, RT-qPCR for EV-associated nucleic acids requires more appropriate housekeeping genes or reference genes, and universal genes that meet all the criteria as control genes for EV-associated nucleic acids have not yet been identified^[152]^. For EV-associated miRNAs, current protocols recommend that samples be processed from aligned volumes and that technical variations should be compensated for using synthetic nonhuman miRNAs, such as *Caenorhabditis elegans* cel-miR-39, as normalization controls. Variations can stem from many sources, such as differences in sample preparation, stabilization, RNA extraction, and target quantification. These differences are not a consequence of the disease state itself. Therefore, optimal genes that are stably expressed, irrespective of the experimental situation or treatment, must be identified to define reference genes for normalizing EV-associated nucleic acid expression. Several housekeeping genes or reference genes have been identified for different native tissues and body fluids, and stable endogenous RNAs have been proposed as internal controls; however, a consensus has not been reached^[152]^. In 2002, Vandesompele *et al*.^[153]^ demonstrated that the common RT-qPCR procedure of using only one control gene induced relatively large errors. In this study, they claimed that ideal single internal control genes do not exist and recommended the use of at least three adequate control genes for calculating a normalization factor^[153]^. This issue with normalizers in gene expression analyses continues to this day, and the same issue is found with protein analyses. Quantification of EV-associated proteins also requires an internal control; however, the most suitable internal control for EV proteins for equivalent amounts of protein has not been identified. Not all EVs contain common EV marker proteins, such as Alix, TSG101, CD9, and CD63^[154]^. Hence, this problem with internal controls is a major problem associated with EV-based liquid biopsy, and further investigation is needed to develop biomarker research.

Despite these several concerns, EV-based liquid biopsy will provide higher sensitivity and speciﬁcity than classical biomarkers due to their stability in body ﬂuids, and new technologies are being developed to solve the current limitations of EV-based liquid biopsy, as mentioned in this manuscript. Translating EV cargo profiles into routine clinical diagnostics would be facilitated by efﬁcient alternatives to EV preparation via ultracentrifugation, such as bioﬂuidic devices for high-throughput analysis. Moreover, extraordinary progress has occurred in analytic technologies, such as MS, HTS, and big-data analysis. These technologies have become irreplaceable and familiar analytical tools for researchers analyzing EV-associated molecules. Indeed, some studies have recommended MS-based methods as an alternative to detect EV protein markers after isolation procedures^[155]^. Further technological development will advance societal implementation of EV-based liquid biopsy for cancer.

## CONCLUSIONS

Useful cancer biomarkers in liquid biopsies are urgently required, and EVs represent a promising resource for cancer biomarkers. The development of technologies is accompanied by novel statistical tools, which can utilize high-dimensional machine learning approaches to analyze big data and provide timely decisions. Thus, the future of EV-based liquid biopsy will be associated with multiple academic fields, such as molecular biology, bioengineering, clinical medicine, machine learning, and statistics. Numerous EV-associated studies will likely enhance the performance of cancer biomarkers in early diagnosis, prognosis, surveillance and treatment. Additionally, recent advances in bioinformatics may demonstrate the biological significance of identified cancer markers, which will provide clues for elucidation of cancer pathophysiology. In conclusion, the development of EV-based liquid biopsy will lead to early diagnosis of fatal cancers and tailor-made treatments for individual patients, and such advancements will extend the healthy human life span and reduce medical costs. Similar to the dramatic changes in our daily lives caused by a microscopic virus, a tiny vesicle may also dramatically advance cancer management.

## DECLARATIONS

### Copyright

© The Author(s) 2021.

## References

[1] Doyle LM, Wang MZ (2019). Overview of Extracellular Vesicles, Their Origin, Composition, Purpose, and
Methods for Exosome Isolation and Analysis. Cells.

[2] Siravegna G, Marsoni S, Siena S, Bardelli A (2017). Integrating Liquid Biopsies into the Management of Cancer. Nat Rev Clin Oncol.

[3] Ashley EA (2016). Towards Precision Medicine. Nat Rev Genet.

[4] Hamburg MA, Collins FS (2010). The path to personalized medicine. N Engl J Med.

[5] Siravegna G, Mussolin B, Venesio T (2019). How liquid biopsies can change clinical practice in
oncology. Ann Oncol.

[6] Bettegowda C, Sausen M, Leary RJ (2014). Detection of circulating tumor DNA in early- and late-stage human
malignancies. Sci Transl Med.

[7] Cheng F, Su L, Qian C (2016). Circulating tumor DNA: a promising biomarker in the liquid biopsy of
cancer. Oncotarget.

[8] Roy S, Hochberg FH, Jones PS (2018). Extracellular vesicles: the growth as diagnostics and therapeutics; a
Survey. J Extracell Vesicles.

[9] Yáñez-Mó M, Siljander PR, Andreu Z (2015). Biological properties of extracellular vesicles and their physiological
functions. J Extracell Vesicles.

[10] Li Y, Zheng Q, Bao C (2015). Circular RNA is enriched and stable in exosomes: a promising biomarker for
cancer diagnosis. Cell Res.

[11] Keller S, Ridinger J, Rupp AK, Janssen JW, Altevogt P (2011). Body fluid derived exosomes as a novel template for clinical
diagnostics. J Transl Med.

[12] Krebs MG, Metcalf RL, Carter L (2014). Molecular analysis of circulating tumour cells - biology and
biomarkers. Nat Rev Clin Oncol.

[13] Diaz LA Jr, Bardelli A (2014). Liquid biopsies: genotyping circulating tumor DNA. J Clin Oncol.

[14] Cristofanilli M, Budd GT, Ellis MJ (2004). Circulating tumor cells, disease progression, and survival in metastatic
breast cancer. N Engl J Med.

[15] de Bono JS, Scher HI, Montgomery RB (2008). Circulating tumor cells predict survival benefit from treatment in
metastatic castration-resistant prostate cancer. Clin Cancer Res.

[16] Danila DC, Heller G, Gignac GA (2007). Circulating tumor cell number and prognosis in progressive
castration-resistant prostate cancer. Clin Cancer Res.

[17] Heller G, McCormack R, Kheoh T (2018). Circulating Tumor Cell Number as a Response Measure of Prolonged Survival
for Metastatic Castration-Resistant Prostate Cancer: A Comparison with Prostate-Specific
Antigen across Five Randomized Phase III Clinical Trials. J Clin Oncol.

[18] Goldkorn A, Ely B, Quinn DI (2014). Circulating tumor cell counts are prognostic of overall survival in SWOG
S0421: a phase III trial of docetaxel with or without atrasentan for metastatic
castration-resistant prostate cancer. J Clin Oncol.

[19] Scher HI, Heller G, Molina A (2015). Circulating tumor cell biomarker panel as an individual-level surrogate for
survival in metastatic castration-resistant prostate cancer. J Clin Oncol.

[20] Allard WJ, Matera J, Miller MC (2004). Tumor cells circulate in the peripheral blood of all major carcinomas but
not in healthy subjects or patients with nonmalignant diseases. Clin Cancer Res.

[21] Alix-Panabières C, Pantel K (2013). Circulating tumor cells: liquid biopsy of cancer. Clin Chem.

[22] Qin J, Alt JR, Hunsley BA, Williams TL, Fernando MR (2014). Stabilization of circulating tumor cells in blood using a collection device
with a preservative reagent. Cancer Cell Int.

[23] Alix-Panabières C, Pantel K (2014). Challenges in circulating tumour cell research. Nat Rev Cancer.

[24] Allard WJ, Terstappen LWMM (2015). CCR 20th anniversary commentary: paving the way for circulating tumor
cells. Clin Cancer Res.

[25] Markou A, Zavridou M, Sourvinou I (2016). Direct comparison of metastasis-related miRNAs expression levels in
circulating tumor cells, corresponding plasma, and primary tumors of breast cancer
patients. Clin Chem.

[26] Antonarakis ES, Lu C, Luber B (2017). Clinical Significance of Androgen Receptor Splice Variant-7 mRNA Detection
in Circulating Tumor Cells of Men With Metastatic Castration-Resistant Prostate Cancer
Treated With First- and Second-Line Abiraterone and Enzalutamide. J Clin Oncol.

[27] Sinkala E, Sollier-Christen E, Renier C (2017). Profiling protein expression in circulating tumour cells using microfluidic
western blotting. Nat Commun.

[28] Armbrecht L, Rutschmann O, Szczerba BM, Nikoloff J, Aceto N, Dittrich PS (2020). Quantification of Protein Secretion from Circulating Tumor Cells in
Microfluidic Chambers. Adv Sci (Weinh).

[29] Zill OA, Banks KC, Fairclough SR (2018). The Landscape of Actionable Genomic Alterations in Cell-Free Circulating
Tumor DNA from 21,807 Advanced Cancer Patients. Clin Cancer Res.

[30] Chun FK, Müller I, Lange I (2006). Circulating tumour-associated plasma DNA represents an independent and
informative predictor of prostate cancer. BJU Int.

[31] Altimari A, Grigioni AD, Benedettini E (2008). Diagnostic role of circulating free plasma DNA detection in patients with
localized prostate cancer. Am J Clin Pathol.

[32] Bergsmedh A, Szeles A, Henriksson M (2001). Horizontal transfer of oncogenes by uptake of apoptotic
bodies. Proc Natl Acad Sci U S A.

[33] Trejo-Becerril C, Pérez-Cárdenas E, Taja-Chayeb L (2012). Cancer progression mediated by horizontal gene transfer in an in vivo
model. PLoS One.

[34] Mahon KL, Qu W, Devaney J, PRIMe consortium (2014). Methylated Glutathione S-transferase 1 (mGSTP1) is a potential plasma free
DNA epigenetic marker of prognosis and response to chemotherapy in castrate-resistant
prostate cancer. Br J Cancer.

[35] Azad AA, Volik SV, Wyatt AW (2015). Androgen Receptor Gene Aberrations in Circulating Cell-Free DNA: Biomarkers
of Therapeutic Resistance in Castration-Resistant Prostate Cancer. Clin Cancer Res.

[36] Lallous N, Volik SV, Awrey S (2016). Functional analysis of androgen receptor mutations that confer
anti-androgen resistance identified in circulating cell-free DNA from prostate cancer
patients. Genome Biol.

[37] Salvi S, Casadio V, Conteduca V (2015). Circulating cell-free AR and CYP17A1 copy number variations may associate
with outcome of metastatic castration-resistant prostate cancer patients treated with
abiraterone. Br J Cancer.

[38] Carreira S, Romanel A, Goodall J (2014). Tumor clone dynamics in lethal prostate cancer. Sci Transl Med.

[39] Diehl F, Schmidt K, Choti MA (2008). Circulating mutant DNA to assess tumor dynamics. Nat Med.

[40] Leung F, Kulasingam V, Diamandis EP (2016). Circulating tumor DNA as a cancer biomarker: fact or
fiction?. Clin Chem.

[41] Sozzi G, Roz L, Conte D (2005). Effects of prolonged storage of whole plasma or isolated plasma DNA on the
results of circulating DNA quantification assays. J Natl Cancer Inst.

[42] Gormally E, Caboux E, Vineis P, Hainaut P (2007). Circulating free DNA in plasma or serum as biomarker of carcinogenesis:
practical aspects and biological significance. Mutat Res.

[43] Cai X, Janku F, Zhan Q, Fan JB (2015). Accessing genetic information with liquid biopsies. Trends Genet.

[44] Schwarzenbach H, Hoon DSB, Pantel K (2011). Cell-free nucleic acids as biomarkers in cancer patients. Nat Rev Cancer.

[45] Gould SJ, Raposo G (2013). As we wait: coping with an imperfect nomenclature for extracellular
vesicles. J Extracell Vesicles.

[46] György B, Szabó TG, Pásztói M (2011). Membrane vesicles, current state-of-the-art: emerging role of extracellular
vesicles. Cell Mol Life Sci.

[47] Théry C, Witwer KW, Aikawa E (2018). Minimal information for studies of extracellular vesicles 2018 (MISEV2018):
a position statement of the International Society for Extracellular Vesicles and update
of the MISEV2014 guidelines. J Extracell Vesicles.

[48] Witwer KW, Théry C (2019). Extracellular vesicles or exosomes?. J Extracell Vesicles.

[49] György B, Szabó TG, Pásztói M (2011). Membrane vesicles, current state-of-the-art: emerging role of extracellular
vesicles. Cell Mol Life Sci.

[50] Raposo G, Stoorvogel W (2013). Extracellular vesicles: exosomes, microvesicles, and
friends. J Cell Biol.

[51] Kowal J, Arras G, Colombo M (2016). Proteomic comparison defines novel markers to characterize heterogeneous
populations of extracellular vesicle subtypes. Proc Natl Acad Sci U S A.

[52] Pan B, Johnstone RM (1983). Fate of the transferrin receptor during maturation of sheep reticulocytes
in vitro: Selective externalization of the receptor. Cell.

[53] Johnstone RM, Adam M, Hammond JR, Orr L, Turbide C (1987). Vesicle formation during reticulocyte maturation. association of plasma
membrane activities with released vesicles (exosomes). J Biol Chem.

[54] Raposo G, Nijman HW, Stoorvogel W (1996). B lymphocytes secrete antigen-presenting vesicles. J Exp Med.

[55] Valadi H, Ekström K, Bossios A, Sjöstrand M, Lee JJ, Lötvall JO (2007). Exosome-mediated transfer of mRNAs and microRNAs is a novel mechanism of
genetic exchange between cells. Nat Cell Biol.

[56] Aebersold R, Mann M (2016). Mass-spectrometric exploration of proteome structure and
function. Nature.

[57] Reuter JA, Spacek DV, Snyder MP (2015). High-throughput sequencing technologies. Mol Cell.

[58] Welch JL, Madison MN, Margolick JB (2017). Effect of prolonged freezing of semen on exosome recovery and biologic
activity. Sci Rep.

[59] Tovar-Camargo OA, Toden S, Goel A (2016). Exosomal microRNA biomarkers: emerging frontiers in colorectal and other
human cancers. Expert Rev Mol Diagn.

[60] Li P, Kaslan M, Lee SH, Yao J, Gao Z (2017). Progress in exosome isolation techniques. Theranostics.

[61] Nordin JZ, Lee Y, Vader P (2015). Ultrafiltration with size-exclusion liquid chromatography for high yield
isolation of extracellular vesicles preserving intact biophysical and functional
properties. Nanomedicine.

[62] Gupta S, Rawat S, Arora V (2018). An improvised one-step sucrose cushion ultracentrifugation method for
exosome isolation from culture supernatants of mesenchymal stem cells. Stem Cell Res Ther.

[63] Van Deun J, Mestdagh P, Sormunen R (2014). The impact of disparate isolation methods for extracellular vesicles on
downstream RNA profiling. J Extracell Vesicles.

[64] Coumans FAW, Brisson AR, Buzas EI (2017). Methodological guidelines to study extracellular vesicles. Circ Res.

[65] Helwa I, Cai J, Drewry MD (2017). A Comparative Study of Serum Exosome Isolation Using Differential
Ultracentrifugation and Three Commercial Reagents. PLoS One.

[66] Clayton A, Court J, Navabi H (2001). Analysis of antigen presenting cell derived exosomes, based on
immuno-magnetic isolation and flow cytometry. J Immunol Methods.

[67] Théry C, Amigorena S, Raposo G, Clayton A (2006). Isolation and characterization of exosomes from cell culture supernatants
and biological fluids. Curr Protoc Cell Biol.

[68] Brennan K, Martin K, FitzGerald SP (2020). A comparison of methods for the isolation and separation of extracellular
vesicles from protein and lipid particles in human serum. Sci Rep.

[69] Liangsupree T, Multia E, Riekkola ML (2021). Modern isolation and separation techniques for extracellular
vesicles. J Chromatogr A.

[70] An M, Wu J, Zhu J, Lubman DM (2018). Comparison of an Optimized Ultracentrifugation Method versus Size-Exclusion
Chromatography for Isolation of Exosomes from Human Serum. J Proteome Res.

[71] Koh YQ, Almughlliq FB, Vaswani K, Peiris HN, Mitchell MD (2018). Exosome enrichment by ultracentrifugation and size exclusion
chromatography. Front Biosci (Landmark Ed).

[72] Wei R, Zhao L, Kong G (2020). Combination of Size-Exclusion Chromatography and Ultracentrifugation
Improves the Proteomic Profiling of Plasma-Derived Small Extracellular
Vesicles. Biol Proced Online.

[73] Simons M, Raposo G (2009). Exosomes - vesicular carriers for intercellular
communication. Curr Opin Cell Biol.

[74] Mathivanan S, Ji H, Simpson RJ (2010). Exosomes: extracellular organelles important in intercellular
communication. J Proteomics.

[75] Kowal J, Tkach M, Théry C (2014). Biogenesis and secretion of exosomes. Curr Opin Cell Biol.

[76] Clayton A, Boilard E, Buzas EI (2019). Considerations towards a roadmap for collection, handling and storage of
blood extracellular vesicles. J Extracell Vesicles.

[77] Jørgensen M, Bæk R, Pedersen S, Søndergaard EK, Kristensen SR, Varming K (2013). Extracellular Vesicle (EV) Array: microarray capturing of exosomes and
other extracellular vesicles for multiplexed phenotyping. J Extracell Vesicles.

[78] Jakobsen KR, Paulsen BS, Bæk R, Varming K, Sorensen BS, Jørgensen MM (2015). Exosomal proteins as potential diagnostic markers in advanced non-small
cell lung carcinoma. J Extracell Vesicles.

[79] Shao H, Chung J, Balaj L (2012). Protein typing of circulating microvesicles allows real-time monitoring of
glioblastoma therapy. Nat Med.

[80] Kravets VG, Schedin F, Jalil R (2013). Singular phase nano-optics in plasmonic metamaterials for label-free
single-molecule detection. Nat Mater.

[81] Im H, Shao H, Park YI (2014). Label-free detection and molecular profiling of exosomes with a
nano-plasmonic sensor. Nat Biotechnol.

[82] Yoshioka Y, Kosaka N, Konishi Y (2014). Ultra-sensitive liquid biopsy of circulating extracellular vesicles using
ExoScreen. Nat Commun.

[83] Zhao Z, Yang Y, Zeng Y, He M (2016). A microfluidic ExoSearch chip for multiplexed exosome detection towards
blood-based ovarian cancer diagnosis. Lab Chip.

[84] Mitchell PJ, Welton J, Staffurth J (2009). Can urinary exosomes act as treatment response markers in prostate
cancer?. J Transl Med.

[85] Logozzi M, Angelini DF, Iessi E (2017). Increased PSA expression on prostate cancer exosomes in in vitro condition
and in cancer patients. Cancer Lett.

[86] Huber V, Fais S, Iero M (2005). Human colorectal cancer cells induce T-cell death through release of
proapoptotic microvesicles: role in immune escape. Gastroenterology.

[87] Khan S, Jutzy JM, Valenzuela MM (2012). Plasma-derived exosomal survivin, a plausible biomarker for early detection
of prostate cancer. PLoS One.

[88] Khan S, Bennit HF, Turay D (2014). Early diagnostic value of survivin and its alternative splice variants in
breast cancer. BMC Cancer.

[89] Melo SA, Luecke LB, Kahlert C (2015). Glypican-1 identifies cancer exosomes and detects early pancreatic
cancer. Nature.

[90] (2017). Book: ISEV2017. J Extracell Vesicles.

[91] Giampieri R, Piva F, Occhipinti G (2019). Clinical impact of different exosomes' protein expression in pancreatic
ductal carcinoma patients treated with standard first line palliative
chemotherapy. PLoS One.

[92] Ning K, Wang T, Sun X (2017). UCH-L1-containing exosomes mediate chemotherapeutic resistance transfer in
breast cancer. J Surg Oncol.

[93] Lv MM, Zhu XY, Chen WX (2014). Exosomes mediate drug resistance transfer in MCF-7 breast cancer cells and
a probable mechanism is delivery of P-glycoprotein. Tumour Biol.

[94] Zhang FF, Zhu YF, Zhao QN (2014). Microvesicles mediate transfer of P-glycoprotein to paclitaxel-sensitive
A2780 human ovarian cancer cells, conferring paclitaxel-resistance. Eur J Pharmacol.

[95] Corcoran C, Rani S, O'Brien K (2012). Docetaxel-resistance in prostate cancer: evaluating associated phenotypic
changes and potential for resistance transfer via exosomes. PLoS One.

[96] Ciravolo V, Huber V, Ghedini GC (2012). Potential role of HER2-overexpressing exosomes in countering
trastuzumab-based therapy. J Cell Physiol.

[97] Martinez VG, O'Neill S, Salimu J (2017). Resistance to HER2-targeted anti-cancer drugs is associated with immune
evasion in cancer cells and their derived extracellular vesicles. Oncoimmunology.

[98] Chen L, Han X (2015). Anti-PD-1/PD-L1 therapy of human cancer: past, present, and
future. J Clin Invest.

[99] Ribas A, Hamid O, Daud A (2016). Association of pembrolizumab with tumor response and survival among
patients with advanced melanoma. JAMA.

[100] Zaretsky JM, Garcia-Diaz A, Shin DS (2016). Mutations Associated with Acquired Resistance to PD-1 Blockade in
Melanoma. N Engl J Med.

[101] Chen G, Huang AC, Zhang W (2018). Exosomal PD-L1 contributes to immunosuppression and is associated with
anti-PD-1 response. Nature.

[102] Cao L, Cui X, Hu J (2017). Advances in digital polymerase chain reaction (dPCR) and its emerging
biomedical applications. Biosens Bioelectron.

[103] Ratajczak J, Miekus K, Kucia M (2006). Embryonic stem cell-derived microvesicles reprogram hematopoietic
progenitors: evidence for horizontal transfer of mRNA and protein
delivery. Leukemia.

[104] Kosaka N, Iguchi H, Yoshioka Y, Takeshita F, Matsuki Y, Ochiya T (2010). Secretory mechanisms and intercellular transfer of microRNAs in living
cells. J Biol Chem.

[105] Pegtel DM, Cosmopoulos K, Thorley-Lawson DA (2010). Functional delivery of viral miRNAs via exosomes. Proc Natl Acad Sci U S A.

[106] Zhang Y, Liu D, Chen X (2010). Secreted monocytic miR-150 enhances targeted endothelial cell
migration. Mol Cell.

[107] Zhu L, Li J, Gong Y (2019). Exosomal tRNA-derived small RNA as a promising biomarker for cancer
diagnosis. Mol Cancer.

[108] Zhang PF, Gao C, Huang XY (2020). Cancer cell-derived exosomal circUHRF1 induces natural killer cell
exhaustion and may cause resistance to anti-PD1 therapy in hepatocellular
carcinoma. Mol Cancer.

[109] Gusachenko ON, Zenkova MA, Vlassov VV (2013). Nucleic acids in exosomes: disease markers and intercellular communication
molecules. Biochem.

[110] Bullock MD, Silva AM, Kanlikilicer-Unaldi P (2015). Exosomal non-coding RNAs: diagnostic, prognostic and therapeutic
applications in cancer. Noncoding RNA.

[111] Mitchell PS, Parkin RK, Kroh EM (2008). Circulating microRNAs as stable blood-based markers for cancer
detection. Proc Natl Acad Sci U S A.

[112] Taylor DD, Gercel-Taylor C (2008). MicroRNA signatures of tumor-derived exosomes as diagnostic biomarkers of
ovarian cancer. Gynecol Oncol.

[113] Rabinowits G, Gerçel-Taylor C, Day JM, Taylor DD, Kloecker GH (2009). Exosomal microRNA: a diagnostic marker for lung cancer. Clin Lung Cancer.

[114] Takeshita N, Hoshino I, Mori M (2013). Serum microRNA expression profile: miR-1246 as a novel diagnostic and
prognostic biomarker for oesophageal squamous cell carcinoma. Br J Cancer.

[115] Nilsson J, Skog J, Nordstrand A (2009). Prostate cancer-derived urine exosomes: a novel approach to biomarkers for
prostate cancer. Br J Cancer.

[116] Foj L, Ferrer F, Serra M (2017). Exosomal and Non-Exosomal Urinary miRNAs in Prostate Cancer Detection and
Prognosis. Prostate.

[117] Yasui T, Yanagida T, Ito S (2017). Unveiling massive numbers of cancer-related urinary-microRNA candidates via
nanowires. Sci Adv.

[118] McKiernan J, Donovan MJ, O'Neill V (2016). A Novel Urine Exosome Gene Expression Assay to Predict High-grade Prostate
Cancer at Initial Biopsy. JAMA Oncol.

[119] Huang X, Yuan T, Liang M (2015). Exosomal miR-1290 and miR-375 as prognostic markers in castration-resistant
prostate cancer. Eur Urol.

[120] Tanaka Y, Kamohara H, Kinoshita K (2013). Clinical impact of serum exosomal microRNA-21 as a clinical biomarker in
human esophageal squamous cell carcinoma. Cancer.

[121] Matsumura T, Sugimachi K, Iinuma H (2015). Exosomal microRNA in serum is a novel biomarker of recurrence in human
colorectal cancer. Br J Cancer.

[122] Işın M, Uysaler E, Özgür E (2015). Exosomal lncRNA-p21 levels may help to distinguish prostate cancer from
benign disease. Front Genet.

[123] Lin LY, Yang L, Zeng Q (2018). Tumor-originated exosomal LncUEGC1 as a circulating biomarker for
early-stage gastric cancer. Mol Cancer.

[124] Zhao R, Zhang Y, Zhang X (2018). Exosomal long noncoding RNA HOTTIP as potential novel diagnostic and
prognostic biomarker test for gastric cancer. Mol Cancer.

[125] Lee YR, Kim G, Tak WY (2019). Circulating exosomal noncoding RNAs as prognostic biomarkers in human
hepatocellular carcinoma. Int J Cancer.

[126] Chen X, Chen RX, Wei WS (2018). PRMT5 Circular RNA Promotes Metastasis of Urothelial Carcinoma of the
Bladder through Sponging miR-30c to Induce Epithelial-Mesenchymal
Transition. Clin Cancer Res.

[127] Ogata-Kawata H, Izumiya M, Kurioka D (2014). Circulating exosomal microRNAs as biomarkers of colon
cancer. PLoS One.

[128] Svedman FC, Lohcharoenkal W, Bottai M (2018). Extracellular microvesicle microRNAs as predictive biomarkers for targeted
therapy in metastastic cutaneous malignant melanoma. PLoS One.

[129] Tang S, Zheng K, Tang Y, Li Z, Zou T, Liu D (2019). Overexpression of serum exosomal HOTAIR is correlated with poor survival
and poor response to chemotherapy in breast cancer patients. J Biosci.

[130] Dong H, Wang W, Chen R (2018). Exosome-mediated transfer of lncRNASNHG14 promotes trastuzumab
chemoresistance in breast cancer. Int J Oncol.

[131] Mikamori M, Yamada D, Eguchi H (2017). MicroRNA-155 Controls Exosome Synthesis and Promotes Gemcitabine Resistance
in Pancreatic Ductal Adenocarcinoma. Sci Rep.

[132] Del Re M, Biasco E, Crucitta S (2017). The Detection of Androgen Receptor Splice Variant 7 in Plasma-derived
Exosomal RNA Strongly Predicts Resistance to Hormonal Therapy in Metastatic Prostate
Cancer Patients. Eur Urol.

[133] Woo HK, Park J, Ku JY (2018). Urine-based liquid biopsy: non-invasive and sensitive AR-V7 detection in
urinary EVs from patients with prostate cancer. Lab Chip.

[134] Del Re M, Marconcini R, Pasquini G (2018). PD-L1 mRNA expression in plasma-derived exosomes is associated with
response to anti-PD-1 antibodies in melanoma and NSCLC. Br J Cancer.

[135] Ma X, Chen Z, Hua D (2014). Essential role for TrpC5-containing extracellular vesicles in breast cancer
with chemotherapeutic resistance. Proc Natl Acad Sci U S A.

[136] Yang SJ, Wang DD, Li J (2017). Predictive role of GSTP1-containing exosomes in chemotherapy-resistant
breast cancer. Gene.

[137] Shao H, Chung J, Lee K (2015). Chip-based analysis of exosomal mRNA mediating drug resistance in
glioblastoma. Nat Commun.

[138] Balaj L, Lessard R, Dai L (2011). Tumour microvesicles contain retrotransposon elements and amplified
oncogene sequences. Nat Commun.

[139] Yokoi A, Villar-Prados A, Oliphint PA (2019). Mechanisms of nuclear content loading to exosomes. Sci Adv.

[140] Thakur BK, Zhang H, Becker A (2014). Double-stranded DNA in exosomes: a novel biomarker in cancer
detection. Cell Res.

[141] Kahlert C, Melo SA, Protopopov A (2014). Identification of double-stranded genomic DNA spanning all chromosomes with
mutated KRAS and p53 DNA in the serum exosomes of patients with pancreatic
cancer. J Biol Chem.

[142] Kahlert C (2019). Liquid Biopsy: Is There an Advantage to Analyzing Circulating Exosomal DNA
Compared to cfDNA or Are They the Same?. Cancer Res.

[143] Allenson K, Castillo J, San Lucas FA (2017). High prevalence of mutant KRAS in circulating exosome-derived DNA from
early-stage pancreatic cancer patients. Ann Oncol.

[144] Bernard V, Kim DU, San Lucas FA (2019). Circulating Nucleic Acids Are Associated With Outcomes of Patients With
Pancreatic Cancer. Gastroenterology.

[145] Castellanos-Rizaldos E, Grimm DG, Tadigotla V Exosome-Based Detection of. EGFR.

[146] Krug AK, Enderle D, Karlovich C (2018). Improved EGFR mutation detection using combined exosomal RNA and
circulating tumor DNA in NSCLC patient plasma. Ann Oncol.

[147] Lee DH, Yoon H, Park S (2018). Urinary Exosomal and cell-free DNA Detects Somatic Mutation and Copy Number
Alteration in Urothelial Carcinoma of Bladder. Sci Rep.

[148] Takahashi A, Okada R, Nagao K (2017). Exosomes maintain cellular homeostasis by excreting harmful DNA from
cells. Nat Commun.

[149] Fischer S, Cornils K, Speiseder T (2016). Indication of Horizontal DNA Gene Transfer by Extracellular
Vesicles. PLoS One.

[150] Jin Y, Chen K, Wang Z (2016). DNA in serum extracellular vesicles is stable under different storage
conditions. BMC Cancer.

[151] Mandel P, Métais P (1948). Les Acides Nucleiques Du Plasma Sanguin Chez l’ Homme. C R Seances Soc Biol Fil.

[152] Schwarzenbach H, da Silva AM, Calin G, Pantel K (2015). Data Normalization Strategies for MicroRNA Quantification. Clin Chem.

[153] Vandesompele J, De Preter K, Pattyn F (2002). Accurate normalization of real-time quantitative RT-PCR data by geometric
averaging of multiple internal control genes. Genome Biol.

[154] Mathivanan S, Simpson RJ (2009). ExoCarta: A compendium of exosomal proteins and RNA. Proteomics.

[155] Nguyen HQ, Lee D, Kim Y (2019). Platelet Factor 4 as a Novel Exosome Marker in MALDI-MS Analysis of
Exosomes from Human Serum. Anal Chem.

[156] Lu Q, Zhang J, Allison R (2009). Identification of extracellular delta-catenin accumulation for prostate
cancer detection. Prostate.

[157] Kharaziha P, Chioureas D, Rutishauser D (2015). Molecular profiling of prostate cancer derived exosomes may reveal a
predictive signature for response to docetaxel. Oncotarget.

[158] Biggs CN, Siddiqui KM, Al-Zahrani AA (2016). Prostate extracellular vesicles in patient plasma as a liquid biopsy
platform for prostate cancer using nanoscale flow cytometry. Oncotarget.

[159] Smalley DM, Sheman NE, Nelson K, Theodorescu D (2008). Isolation and identification of potential urinary microparticle biomarkers
of bladder cancer. J Proteome Res.

[160] Chen CL, Lai YF, Tang P (2012). Comparative and targeted proteomic analyses of urinary microparticles from
bladder cancer and hernia patients. J Proteome Res.

[161] Lin SY, Chang CH, Wu HC (2016). Proteome Profiling of Urinary Exosomes Identifies Alpha 1-Antitrypsin and
H2B1K as Diagnostic and Prognostic Biomarkers for Urothelial Carcinoma. Sci Rep.

[162] Silvers CR, Miyamoto H, Messing EM, Netto GJ, Lee YF (2017). Characterization of urinary extracellular vesicle proteins in
muscle-invasive bladder cancer. Oncotarget.

[163] Silvers CR, Liu YR, Wu CH, Miyamoto H, Messing EM, Lee YF (2016). Identification of extracellular vesicle-borne periostin as a feature of
muscle-invasive bladder cancer. Oncotarget.

[164] Beckham CJ, Olsen J, Yin PN (2014). Bladder cancer exosomes contain EDIL-3/Del1 and facilitate cancer
progression. J Urol.

[165] Welton JL, Khanna S, Giles PJ (2010). Proteomics analysis of bladder cancer exosomes. Mol Cell Proteomics.

[166] Raimondo F, Morosi L, Corbetta S (2013). Differential protein profiling of renal cell carcinoma urinary
exosomes. Mol Biosyst.

[167] Wang X, Zhong W, Bu J (2019). Exosomal protein CD82 as a diagnostic biomarker for precision medicine for
breast cancer. Mol Carcinog.

[168] Rupp AK, Rupp C, Keller S (2011). Loss of EpCAM expression in breast cancer derived serum exosomes: role of
proteolytic cleavage. Gynecol Oncol.

[169] Wang T, Ning K, Lu TX (2017). Increasing circulating exosomes-carrying TRPC5 predicts chemoresistance in
metastatic breast cancer patients. Cancer Sci.

[170] Szajnik M, Derbis M, Lach M (2013). Exosomes in Plasma of Patients with Ovarian Carcinoma: Potential Biomarkers
of Tumor Progression and Response to Therapy. Gynecol Obstet (Sunnyvale).

[171] Li J, Sherman-Baust CA, Tsai-Turton M, Bristow RE, Roden RB, Morin PJ (2009). Claudin-containing exosomes in the peripheral circulation of women with
ovarian cancer. BMC Cancer.

[172] Tang MKS, Yue PYK, Ip PP (2018). Soluble E-cadherin promotes tumor angiogenesis and localizes to exosome
surface. Nat Commun.

[173] Graves LE, Ariztia EV, Navari JR, Matzel HJ, Stack MS, Fishman DA (2004). Proinvasive properties of ovarian cancer ascites-derived membrane
vesicles. Cancer Res.

[174] Keller S, König AK, Marmé F (2009). Systemic presence and tumor-growth promoting effect of ovarian carcinoma
released exosomes. Cancer Lett.

[175] Frampton AE, Prado MM, López-Jiménez E (2018). Glypican-1 is enriched in circulating-exosomes in pancreatic cancer and
correlates with tumor burden. Oncotarget.

[176] Kimura H, Yamamoto H, Harada T (2019). CKAP4, a DKK1 Receptor, Is a Biomarker in Exosomes Derived from Pancreatic
Cancer and a Molecular Target for Therapy. Clin Cancer Res.

[177] Costa-Silva B, Aiello NM, Ocean AJ (2015). Pancreatic cancer exosomes initiate pre-metastatic niche formation in the
liver. Nat Cell Biol.

[178] Yokoyama S, Takeuchi A, Yamaguchi S (2017). Clinical implications of carcinoembryonic antigen distribution in serum
exosomal fraction-Measurement by ELISA. PLoS One.

[179] Choi DS, Park JO, Jang SC (2011). Proteomic analysis of microvesicles derived from human colorectal cancer
ascites. Proteomics.

[180] Baran J, Baj-Krzyworzeka M, Weglarczyk K (2010). Circulating tumour-derived microvesicles in plasma of gastric cancer
patients. Cancer Immunol Immunother.

[181] Yamashita T, Kamada H, Kanasaki S (2013). Epidermal growth factor receptor localized to exosome membranes as a
possible biomarker for lung cancer diagnosis. Pharmazie.

[182] Niu L, Song X, Wang N, Xue L, Song X, Xie L (2019). Tumor-derived exosomal proteins as diagnostic biomarkers in non-small cell
lung cancer. Cancer Sci.

[183] Sandfeld-Paulsen B, Aggerholm-Pedersen N, Bæk R (2016). Exosomal proteins as prognostic biomarkers in non-small cell lung
cancer. Mol Oncol.

[184] Li Y, Zhang Y, Qiu F, Qiu Z (2011). Proteomic identification of exosomal LRG1: a potential urinary biomarker
for detecting NSCLC. Electrophoresis.

[185] Logozzi M, De Milito A, Lugini L (2009). High levels of exosomes expressing CD63 and caveolin-1 in plasma of
melanoma patients. PLoS One.

[186] Peinado H, Alečković M, Lavotshkin S (2012). Melanoma exosomes educate bone marrow progenitor cells toward a
pro-metastatic phenotype through MET. Nat Med.

[187] Bryant RJ, Pawlowski T, Catto JW (2012). Changes in circulating microRNA levels associated with prostate
cancer. Br J Cancer.

[188] Xu Y, Qin S, An T, Tang Y, Huang Y, Zheng L (2017). MiR-145 detection in urinary extracellular vesicles increase diagnostic
efficiency of prostate cancer based on hydrostatic filtration dialysis
method. Prostate.

[189] Koppers-Lalic D, Hackenberg M, de Menezes R (2016). Noninvasive prostate cancer detection by measuring miRNA variants (isomiRs)
in urine extracellular vesicles. Oncotarget.

[190] Wani S, Kaul D, Mavuduru RS, Kakkar N, Bhatia A (2017). Urinary-exosomal miR-2909: A novel pathognomonic trait of prostate cancer
severity. J Biotechnol.

[191] Rodríguez M, Bajo-Santos C, Hessvik NP (2017). Identification of non-invasive miRNAs biomarkers for prostate cancer by
deep sequencing analysis of urinary exosomes. Mol Cancer.

[192] Endzeliņš E, Berger A, Melne V (2017). Detection of circulating miRNAs: comparative analysis of extracellular
vesicle-incorporated miRNAs and cell-free miRNAs in whole plasma of prostate cancer
patients. BMC Cancer.

[193] Li Z, Ma YY, Wang J (2016). Exosomal microRNA-141 is upregulated in the serum of prostate cancer
patients. Onco Targets Ther.

[194] Hessvik NP, Sandvig K, Llorente A (2013). Exosomal miRNAs as Biomarkers for Prostate Cancer. Front Genet.

[195] Moltzahn F, Olshen AB, Baehner L (2011). Microfluidic-based multiplex qRT-PCR identifies diagnostic and prognostic
microRNA signatures in the sera of prostate cancer patients. Cancer Res.

[196] Lodes MJ, Caraballo M, Suciu D, Munro S, Kumar A, Anderson B (2009). Detection of cancer with serum miRNAs on an oligonucleotide
microarray. PLoS One.

[197] Bhagirath D, Yang TL, Bucay N (2018). microRNA-1246 Is an Exosomal Biomarker for Aggressive Prostate
Cancer. Cancer Res.

[198] Wang YH, Ji J, Wang BC (2018). Tumor-Derived Exosomal Long Noncoding RNAs as Promising Diagnostic
Biomarkers for Prostate Cancer. Cell Physiol Biochem.

[199] De Long J, Sullivan TB, Humphrey J (2015). A non-invasive miRNA based assay to detect bladder cancer in cell-free
urine. Am J Transl Res.

[200] Armstrong DA, Green BB, Seigne JD, Schned AR, Marsit CJ (2015). MicroRNA molecular profiling from matched tumor and bio-fluids in bladder
cancer. Mol Cancer.

[201] Andreu Z, Otta Oshiro R, Redruello A (2017). Extracellular vesicles as a source for non-invasive biomarkers in bladder
cancer progression. Eur J Pharm Sci.

[202] Baumgart S, Meschkat P, Edelmann P (2018). MP78-05 INVASION-ASSOCIATED MIRNAS S AS POSSIBLE DIAGNOSTIC BIOMARKERS OF
MUSCLE INVASIVE BLADDER CANCER IN TUMOR TISSUES AND URINARY EXOSOMES. J Urol.

[203] Berrondo C, Flax J, Kucherov V (2016). Expression of the Long Non-Coding RNA HOTAIR Correlates with Disease
Progression in Bladder Cancer and Is Contained in Bladder Cancer Patient Urinary
Exosomes. PLoS One.

[204] Zhang S, Du L, Wang L (2019). Evaluation of serum exosomal LncRNA-based biomarker panel for diagnosis and
recurrence prediction of bladder cancer. J Cell Mol Med.

[205] Xue M, Chen W, Xiang A (2017). Hypoxic exosomes facilitate bladder tumor growth and development through
transferring long non-coding RNA-UCA1. Mol Cancer.

[206] Zhang W, Ni M, Su Y (2018). MicroRNAs in Serum Exosomes as Potential Biomarkers in Clear-cell Renal
Cell Carcinoma. Eur Urol Focus.

[207] Wang X, Wang T, Chen C Serum exosomal miR-210 as a potential biomarker for clear cell renal cell
carcinoma. J Cell Biochem.

[208] Butz H, Nofech-Mozes R, Ding Q (2016). Exosomal MicroRNAs Are Diagnostic Biomarkers and Can Mediate Cell-Cell
Communication in Renal Cell Carcinoma. Eur Urol Focus.

[209] Fujii N, Hirata H, Ueno K (2017). Extracellular MiR-224 as a prognostic marker for clear cell renal cell
carcinoma. Oncotarget.

[210] Du M, Giridhar KV, Tian Y (2017). Plasma exosomal miRNAs-based prognosis in metastatic kidney
cancer. Oncotarget.

[211] De Palma G, Sallustio F, Curci C (2016). The Three-Gene Signature in Urinary Extracellular Vesicles from Patients
with Clear Cell Renal Cell Carcinoma. J Cancer.

[212] Hannafon BN, Trigoso YD, Calloway CL (2016). Plasma exosome microRNAs are indicative of breast cancer. Breast Cancer Res.

[213] Zhou W, Fong MY, Min Y (2014). Cancer-secreted miR-105 destroys vascular endothelial barriers to promote
metastasis. Cancer Cell.

[214] Rodríguez-Martínez A, de Miguel-Pérez D, Ortega FG (2019). Exosomal miRNA profile as complementary tool in the diagnostic and
prediction of treatment response in localized breast cancer under neoadjuvant
chemotherapy. Breast Cancer Res.

[215] Santos JC, Lima NDS, Sarian LO, Matheu A, Ribeiro ML, Derchain SFM (2018). Exosome-mediated breast cancer chemoresistance via miR-155
transfer. Sci Rep.

[216] Gregory PA, Bert AG, Paterson EL (2008). The miR-200 family and miR-205 regulate epithelial to mesenchymal
transition by targeting ZEB1 and SIP1. Nat Cell Biol.

[217] Pigati L, Yaddanapudi SC, Iyengar R (2010). Selective release of microRNA species from normal and malignant mammary
epithelial cells. PLoS One.

[218] Kanlikilicer P, Bayraktar R, Denizli M (2018). Exosomal miRNA confers chemo resistance via targeting Cav1/p-gp/M2-type
macrophage axis in ovarian cancer. EBioMedicine.

[219] Que R, Ding G, Chen J, Cao L (2013). Analysis of serum exosomal microRNAs and clinicopathologic features of
patients with pancreatic adenocarcinoma. World J Surg Oncol.

[220] Lau C, Kim Y, Chia D (2013). Role of pancreatic cancer-derived exosomes in salivary biomarker
development. J Biol Chem.

[221] Tian XP, Wang CY, Jin XH (2019). Acidic Microenvironment Up-Regulates Exosomal miR-21 and miR-10b in
Early-Stage Hepatocellular Carcinoma to Promote Cancer Cell Proliferation and
Metastasis. Theranostics.

[222] Xie M, Yu T, Jing X (2020). Exosomal circSHKBP1 promotes gastric cancer progression via regulating the
miR-582-3p/HUR/VEGF axis and suppressing HSP90 degradation. Mol Cancer.

[223] Liu C, Eng C, Shen J (2016). Serum exosomal miR-4772-3p is a predictor of tumor recurrence in stage II
and III colon cancer. Oncotarget.

[224] Bjørnetrø T, Redalen KR, Meltzer S (2019). An experimental strategy unveiling exosomal microRNAs 486-5p, 181a-5p and
30d-5p from hypoxic tumour cells as circulating indicators of high-risk rectal
cancer. J Extracell Vesicles.

[225] Cazzoli R, Buttitta F, Di Nicola M (2013). microRNAs derived from circulating exosomes as noninvasive biomarkers for
screening and diagnosing lung cancer. J Thorac Oncol.

[226] Silva J, García V, Zaballos Á (2011). Vesicle-related microRNAs in plasma of nonsmall cell lung cancer patients
and correlation with survival. Eur Respir J.

[227] Aushev VN, Zborovskaya IB, Laktionov KK (2013). Comparisons of microRNA patterns in plasma before and after tumor removal
reveal new biomarkers of lung squamous cell carcinoma. PLoS One.

[228] Wei F, Ma C, Zhou T (2017). Exosomes derived from gemcitabine-resistant cells transfer malignant
phenotypic traits via delivery of miRNA-222-3p. Mol Cancer.

[229] Yuwen DL, Sheng BB, Liu J, Wenyu W, Shu YQ (2017). MiR-146a-5p level in serum exosomes predicts therapeutic effect of
cisplatin in non-small cell lung cancer. Eur Rev Med Pharmacol Sci.

[230] Shi L, Chen J, Yang J, Pan T, Zhang S, Wang Z (2010). MiR-21 protected human glioblastoma U87MG cells from chemotherapeutic drug
temozolomide induced apoptosis by decreasing Bax/Bcl-2 ratio and caspase-3
activity. Brain Res.

[231] Akers JC, Ramakrishnan V, Kim R (2013). MiR-21 in the extracellular vesicles (EVs) of cerebrospinal fluid (CSF): a
platform for glioblastoma biomarker development. PLoS One.

[232] Manterola L, Guruceaga E, Gállego Pérez-Larraya J (2014). A small noncoding RNA signature found in exosomes of GBM patient serum as a
diagnostic tool. Neuro Oncol.

